# Changes in the chemical defenses of an invasive toad indicate drivers and limitations of adaptation

**DOI:** 10.1016/j.isci.2026.115401

**Published:** 2026-03-25

**Authors:** Max Mühlenhaupt, James Baxter-Gilbert, Julia L. Riley, Buyisile G. Makhubo, Nhlanhla S. Dludla, Cláudia Baider, F.B. Vincent Florens, Xavier Porcel, André de Villiers, Willem A.L. van Otterlo, John Measey

**Affiliations:** 1 Department of Behavioural Ecology, Bielefeld University, Konsequenz 45, 33615 Bielefeld, Germany; 2 Institute of Biology, Freie Universität Berlin, Königin-Luise-Str. 1-3, 14195 Berlin, Germany; 3 Centre for Invasion Biology, Department of Botany and Zoology, Stellenbosch University, Stellenbosch, Western Cape 7600, South Africa; 4 Department of Biology, Mount Allison University, 62 York Street, Sackville, New Brunswick E4L 1G7, Canada; 5 School of Life Sciences, University of KwaZulu-Natal, Private Bag X54001, Durban 40001, South Africa; 6 The Mauritius Herbarium, Agriculture Services, Ministry of Agro-Industry, Food Security and Blue Economy and Fisheries, Le Réduit, Mauritius; 7 Tropical Island Biodiversity, Ecology and Conservation Pole of Research, Faculty of Science, University of Mauritius, Le Réduit, Mauritius; 8 CIBIO, Centro de Investigação em Biodiversidade e Recursos Genéticos, InBIO Laboratório Associado, Universidade do Porto, Vairão, Portugal; 9 Department of Chemistry and Polymer Science, Stellenbosch University, Stellenbosch 7600, South Africa; 10 Centre for Invasion Biology, Institute of Biodiversity, Yunnan University in Kunming, Kunming, Yunnan, China

**Keywords:** Ecology, Environmental science, Evolutionary ecology

## Abstract

Chemical defenses of invasive species provide protection against predators, competitors, and parasites enabling successful colonization of novel environments and can be highly detrimental to native biota. Here, we study how the chemical defenses of a successful invasive toad have changed by comparing the native source population with the introduced populations on Mauritius, Réunion, and in Cape Town. In line with the enemy release hypothesis, on Mauritius and Réunion where the community of predator species is less diverse, the toads show marked reductions in toxin gland size as well as changes in the composition of the toxins. However, in Cape Town, where the introduction happened more recently and the predator community is more diverse, only the composition of the toad toxins changed. Our results indicate drivers and constraints of adaptations of chemical defenses and provide evidence for the role of enemy release in the success of an invasive toad.

## Highlights

• Reduced investment into chemical defenses in line with enemy release hypothesis
• Changes in parotoid gland size more pronounced than changes in toxin composition
• Extent of change correlates with predator diversity and time since colonization
• 15 compounds tentatively identified as toxic components in secretion

## Graphical abstract

**Figure:**
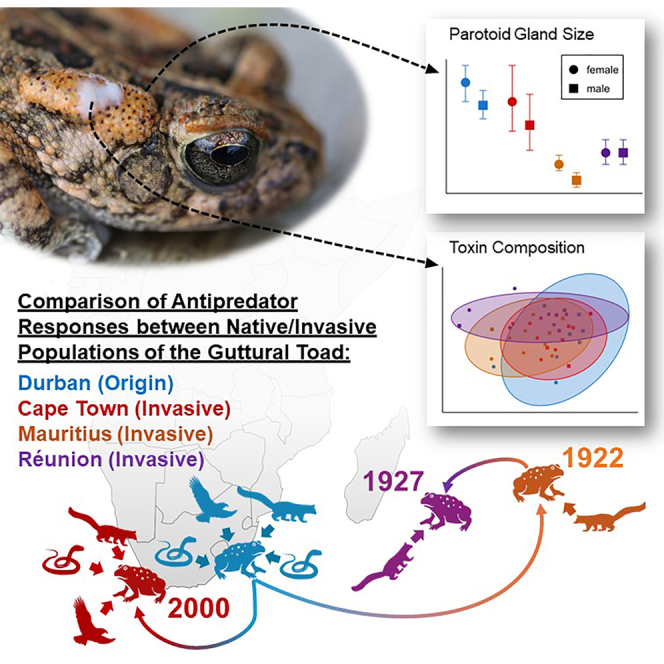

## Introduction

Chemical defenses are highly diverse and complex traits that have evolved in a multitude of lifeforms to avoid predation, herbivory, infection, and competition.^1^^,^^2^ Therefore, they also play an important role in the ability of an organism to respond to rapid changes in its environment that, in many cases, alter the composition of the biological community.^3^ Chemical defenses are known to aid native populations in persisting after the introduction of non-native species^4^ or after anthropogenic modifications to habitats, such as urbanization.^5^^,^^6^^,^^7^ Likewise, many organisms introduced to non-native habitats by human activity rely on chemical defenses to persist in the novel biological community as chemical defenses provide effective protection against predators and parasites that do not share a co-evolutionary history with the non-native species.^3^^,^^8^^,^^9^^,^^10^ These specific defenses can therefore improve competitive ability in the novel environment.^11^^,^^12^ Chemical defenses, however, also incur costs such as metabolic requirements,^13^^,^^14^ trade-offs with other tissues and functions,^15^^,^^16^ or if the chemical defense involves compounds that have to be sequestered from the environment, involve opportunity costs and trade-offs (e.g., food sources for sequestering toxins might represent low quality diets compared to food sources that do not provide toxic compounds^17^^,^^18^). Therefore, organisms facing an altered or novel biological environment will need to re-adjust their chemical defenses to address these trade-offs within the new selective environment.^8^

The capacity for non-native populations to adaptively re-balance energy investment based on altered selective pressures within a novel ecosystem can be understood within the context of the enemy release hypothesis.^19^ It proposes that introduced populations benefit from the release of co-evolved predators and parasites directly (e.g., by reduced mortality due to predation) and indirectly (e.g., by reducing their investment into defensive traits).^19^^,^^20^ Reduced investment into defensive traits might allow higher allocation of resources into growth, dispersal, reproduction, and other traits that can provide a competitive advantage over the native biota in the introduced range.^21^ For example, a meta-analysis focusing on the trade-off between herbivore resistance traits and fitness traits in non-native plant species found that herbivores performed better on non-native populations, while fitness of the non-native populations was higher compared to native congeneric populations.^22^ However, given the complex nature of chemical defenses, the opposite can also be true. Invasive populations can also show increased chemical defenses compared to native populations when these defenses provide a benefit against novel predators^23^ or competitors.^11^ This inherent complexity of chemical defenses due to the dependency on a specific selecting agent (e.g., a specialized predator) highlights the importance of studying the relationship between the novel biotic community and the chemical defenses of introduced populations. A better understanding would help us to improve our predictions of the ability of non-native organisms to persist in novel habitats and their impact in these habitats.

Another important, but often overlooked, feature of chemical defenses and their trajectory of change in invasive populations is the dual nature of such defensive traits. Poisons, venoms, and other toxic secretions can change in quality (i.e., the chemical composition of the secretions) and quantity (i.e., the volume of the secretion produced and exuded).^5^^,^^24^ Changes in both of these two aspects have important fitness consequences as the production of lower potency compounds is likely energetically cheaper, but can also be less effective against certain predators.^25^ Similarly, reducing the quantitative dimensions of the chemical defense saves on the costs of the defense but weakens the overall strength of it.^2^^,^^15^ Therefore, studying divergence in the chemical defenses of invasive populations should incorporate both the quality and quantity of the defense agent. To our knowledge, only in one study, both aspects of chemical defense in invasive populations have been considered (^26^, but see^27^ who identified quantitative and qualitative differences across ontogeny). The focus in this study, however, was on the concentration of only one compound. This approach would limit our insight into the changes of these defenses in response to a novel selective environment as most species implement a mixture of many compounds as their chemical defense.^1^^,^^27^^,^^28^^,^^29^^,^^30^^,^^31^

**Figure 1:**
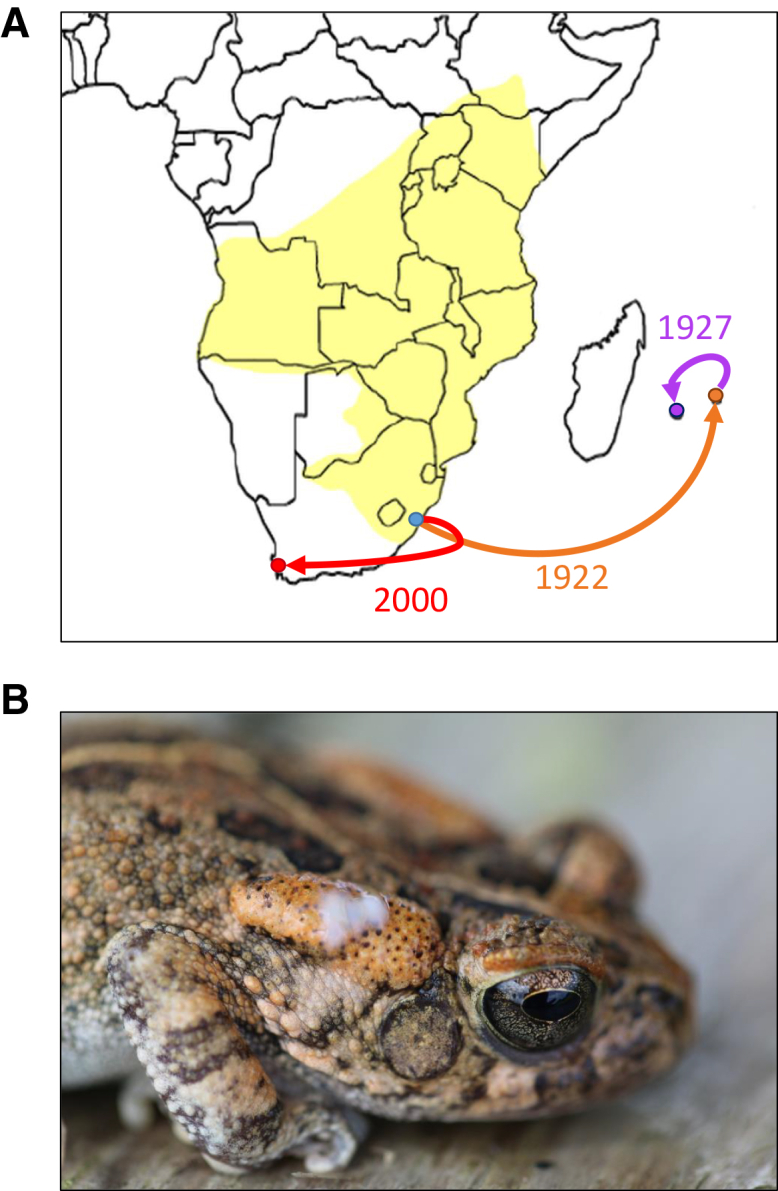
Invasion pathway of the Guttural Toad (*Sclerophrys gutturalis*) (A) The Guttural Toad has a large native distribution in continental Africa (highlighted in yellow). In 1922, Guttural Toads were shipped from the harbor city Durban, South Africa (blue dot) to Mauritius (orange dot) in a futile attempt to use the toads as a biocontrol agent against invertebrate pests.^46^^,^^53^ From Mauritius, adult toads were brought to Réunion (purple dot) five years later. With a generation time of two years and a maximum lifespan of eight years, it is therefore likely that many of the toads brought to Réunion were born in Durban or represent the first generation of toads born on Mauritius. In 2000, Guttural Toads were first heard calling in a peri-urban area of Cape Town (red dot), South Africa. This time, the introduction was likely inadvertently in the form of eggs and tadpoles within a shipment of aquatic vegetation from Durban.^45^^,^^46^ (B) The prominent parotoid gland on the side of the head of a Guttural Toad collected in Mauritius. When attacked, the toads exude a white poisonous secretion from these glands as can be seen in the photo.

Amphibians are well known for their abundant and complex chemical defense mechanisms.^32^^,^^33^ Toads (Bufonidae) represent a family of amphibians that is characterized by the existence of glands across their skin that produce and exude potent cardiotoxins: bufadienolides, also known as “bufotoxins”.^34^^,^^35^^,^^36^ The most prominent skin glands of toads are the parotoid glands, a pair of large glands located behind the eyes. Parotoid glands are primarily responsible for producing, storing, and exuding the toxic secretions of toads.^37^ Therefore, the parotoid gland size correlates with the amount of toxin produced by an individual.^38^^,^^39^ Toads, such as the Cane Toad (*Rhinella marina*) and the Asian Black-spined Toad (*Duttaphrynus melanostictus*), are infamous invasive species that can thrive in a range of non-native habitats and thereby frequently poison the fauna in the ecosystem that they have invaded.^40^^,^^41^^,^^42^^,^^43^ Furthermore, a phylogenetic analysis indicated that the possession of parotoid glands likely enhanced the range-expansion abilities of members of the family Bufonidae.^44^ Another highly invasive toad is the Guttural Toad (*Sclerophrys gutturalis*, hereafter referred to as Guttural Toad (see also^45^^,^^46^^,^^47^^,^^48^^,^^49^^,^^50^) with its native distribution in sub-Saharan Africa and three established invasive populations on Mauritius and Réunion and near Cape Town, South Africa.^45^ All three of these invasive population originated from the same clade (Figure 1),^46^ which makes the Guttural Toad an interesting model system to study how chemical defenses change in different environments and on different time-scales (i.e., approx. 100 years in Mauritius and Réunion and approx. 25 years in Cape Town). These invasive populations show remarkable adaptability in response to the climatic and biotic conditions experienced in the novel ranges that involve physiological, behavioral, and morphological changes.^47^^,^^48^^,^^49^^,^^50^ Importantly, in comparison to the source population, invasive Guttural Toads from Mauritius and Réunion show marked reductions in body size that exceed any reported natural anuran island dwarfism.^50^^,^^51^^,^^52^ Furthermore, the toads from Mauritius and Réunion have developed shorter relative limb sizes and show decreased endurance capacities and escape speeds.^50^^,^^52^ These trait changes could be the result of a relaxed predator regime on the islands; however, no study so far has considered the toad’s chemical defenses.

Here, we compare the size of the parotoid glands and the chemical composition of the secretions from these glands between invasive Guttural Toads and conspecifics from their region of origin to assess changes in quantity and quality of the chemical defenses in response to novel environments experienced by the invasive populations. Given the well-documented link between parotoid gland size and volume of toxic secretion, we examined both the parotoid gland size as well as the composition of the toxic secretion to receive an estimation of the total toxin load a predator would be facing within a given population. Assuming reductions in predator pressure experienced by the invasive populations, we expected smaller parotoid glands and changes in toxin composition in invasive toads compared to native toads (i.e., aligning with the enemy release hypothesis^19^). We also investigated sexual dimorphisms as previous studies have shown sex-specific differences in chemical defenses that are relevant to the ecology of the toad species studied.^6^^,^^23^

## Results

### Differences in parotoid gland size

**Figure 2:**
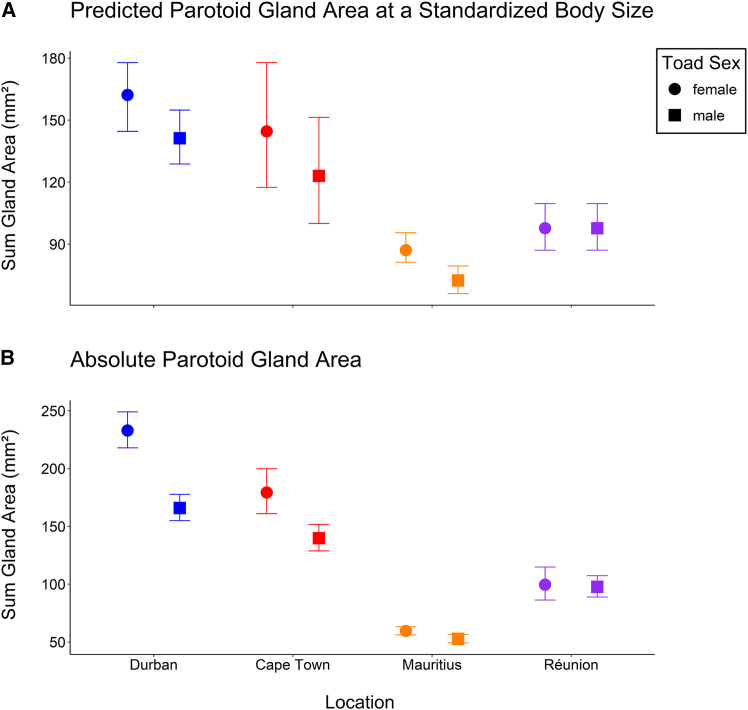
Parotoid gland size (A) The predicted parotoid gland area for female (circle) and male (square) toads from each studied location (Durban—blue, Cape Town—red, Mauritius—orange, and Réunion—violet) based on our linear mixed effects model including log_10_-SVL. Presented are means with their corresponding upper and lower 95% confidence intervals calculated using *emmeans*.^54^ Predictions were made for a toad with an SVL of 62.908 mm, which represents the overall average for toads included in the data. (B) The absolute parotoid gland area of females and males from each studied location. Presented are means with their corresponding 95% confidence intervals (using the *t*-distribution with *n*-1 degrees of freedom).

Parotoid gland size and snout-vent length (SVL) were strongly positively associated (Table 1A). Relative to body size, both female and male toads from Mauritius and Réunion had smaller parotoid glands than toads of the respective sex from Durban, while there was no difference in relative parotoid gland size between males and females from Cape Town in comparison with males and females from Durban (Table 1B and C, Figure 2A). Female toads from Mauritius and Réunion had 46.3% and 39.7% smaller parotoid glands relative to body size than females from Durban, respectively. Similarly, males from Mauritius and Réunion had 48.7% and 30.8% smaller parotoid glands relative to body size than males from Durban, respectively. These changes in parotoid gland size relative to body size exceed the changes in body size documented in Guttural Toads from Mauritius and Réunion.^50^ In all locations but Réunion, female toads had larger parotoid glands relative to body size than male toads (Tables 1B and 1C). Given the greater reduction of relative parotoid gland size of female compared to male toads from Réunion (Table 1B), the loss of the sexual relative parotoid gland size dimorphism in Réunion can be explained by a disproportionately stronger reduction of the relative parotoid gland size of females compared to males. For any other differences between locations and sexes see Table 1.

**Table 1:** Outcome of a linear mixed effects model investigating differences in relative parotoid gland size between locations and sexe**s**

**(A) Model summary**				

Variable Names	–	–	–	–
*Fixed Effects*	*β*	*SE*	*t*	*p*
**Intercept (Cape Town, Female)**	**−0.950**	**0.132**	**−7.185**	**<0.001**
**log**_**10**_**SVL**	**1.74**	**0.069**	**25.372**	**<0.001**
Location (Durban)	0.044	0.044	1.003	0.337
**Location (Mauritius)**	**−0.219**	**0.044**	**−4.974**	**<0.001**
**Location (Réunion)**	**−0.173**	**0.045**	**−3.818**	**0.003**
**Sex (Male)**	**−0.073**	**0.021**	**−3.528**	**<0.001**
Location:Sex (Durban, Male)	0.018	0.026	0.690	0.490
Location:Sex (Mauritius, Male)	−0.012	0.026	−0.480	0.632
**Location:Sex (Réunion, Male)**	**0.071**	**0.027**	**2.619**	**0.009**
*Random Effects*	*σ*^*2*^	*–*	*–*	*–*
Intercept (Site)	0.001	–	–	–
Residuals	0.010	–	–	–

**(B) Multiple comparisons between locations**				

Contrasts	*β*	*SE*	*t*	*p*_*adj*_
Female: Cape Town vs. Durban	−0.044	0.044	−1.00	0.751
**Female: Cape Town vs. Mauritius**	**0.219**	**0.044**	**4.969**	**0.002**
**Female: Cape Town vs. Réunion**	**0.173**	**0.045**	**3.812**	**0.014**
**Female: Durban vs. Mauritius**	**0.263**	**0.029**	**9.121**	**<0.001**
**Female: Durban vs. Réunion**	**0.217**	**0.030**	**7.189**	**<0.001**
Female: Mauritius vs. Réunion	−0.046	0.030	−1.520	0.447
Male: Cape Town vs. Durban	−0.062	0.044	−1.402	0.524
**Male: Cape Town vs. Mauritius**	**0.231**	**0.044**	**5.246**	**0.001**
Male: Cape Town vs. Réunion	0.102	0.046	2.237	0.176
**Male: Durban vs. Mauritius**	**0.293**	**0.029**	**10.255**	**<0.001**
**Male: Durban vs. Réunion**	**0.16**	**0.031**	**5.311**	**<0.001**
**Male: Mauritius vs. Réunion**	**−0.129**	**0.030**	**−4.256**	**0.002**

**(C) Multiple comparisons between sexes**				

Contrasts	*β*	*SE*	*t*	*p*_*adj*_
**Cape Town: Females vs. Males**	**0.073**	**0.021**	**3.528**	**<0.001**
**Durban: Females vs. Males**	**0.055**	**0.016**	**3.38**	**<0.001**
**Mauritius: Females vs. Males**	**0.086**	**0.016**	**5.649**	**<0.001**
Réunion: Females vs. Males	0.002	0.018	0.116	0.908

(A) The table gives coefficient estimates (*β*) that are representative of log_10_-transformed morphological measures with their corresponding standard errors (*SE*) and *t* values. Variance estimates (*σ*^*2*^) are supplied for residuals and random effects. All significant values (*p* < 0.05) are bolded. Reference levels for each categorical variable are supplied in parentheses following the variable name. (B) Post-hoc multiple comparisons of relative parotoid gland size between toads of the same sex and different locations. (C) Post-hoc multiple comparisons investigating sexual relative parotoid gland size dimorphisms in toads of the same location. In multiple comparisons, *p* values were Tukey-adjusted. All significant values (*p* < 0.05) are bolded.

Without standardization for body size, females from Mauritius and Réunion had smaller parotoid glands than females from Durban, while there was no difference between females from Cape Town and Durban in absolute parotoid gland size (Table 2B, Figure 2B). Considering males, absolute parotoid gland size was lower in Mauritius compared to Durban (Table 2B, Figure 2B). The difference in absolute parotoid gland size between males from Réunion and males from Durban was non-significant (*p*_*adj*_ = 0.062) and absolute parotoid gland size between males from Cape Town and Durban was not different (Table 2B). Compared to native female toads from Durban, invasive female toads from Mauritius and Réunion had 74.4% and 57.3% smaller parotoid glands (ignoring body size differences), respectively. On Mauritius, male toads had 68.2% smaller parotoid glands compared to males from Durban and, albeit marginally non-significant (see above, Table 2B), male toads from Réunion had 41.1% smaller parotoid glands than males from Durban. There was a sexual parotoid gland size dimorphism in toads from all locations, with females having larger parotoid glands than males when ignoring the effect of body size (Table 2C, Figure 2B). All other differences are presented in Table 2.

**Table 2:** Outcome of a linear mixed effects model investigating differences in absolute parotoid gland size between locations and sexe**s**

**(A) Model summary**				

Variable Names	–	–	–	–
*Fixed Effects*	*β*	*SE*	*t*	*p*
**Intercept (Cape Town, Female)**	**2.25**	**0.010**	**22.558**	**<0.001**
Location (Durban)	0.105	0.109	0.962	0.355
**Location (Mauritius)**	**−0.445**	**0.108**	**−4.125**	**0.001**
Location (Réunion)	−0.192	0.113	−1.697	0.116
**Sex (Male**	**−0.108**	**0.030**	**−3.656**	**<0.001**
Location:Sex (Durban, Male)	−0.048	0.037	−1.300	0.194
Location:Sex (Mauritius, Male)	0.054	0.037	1.474	0.141
Location:Sex (Réunion, Male)	0.056	0.039	1.423	0.155
*Random Effects*	*σ*^*2*^	*–*	*–*	*–*
Intercept (Site)	0.001	–	–	–
Residuals	0.020	–	–	–

**(B) Multiple comparisons between locations**				

Contrasts	*β*	*SE*	*t*	*p*_*adj*_
Female: Cape Town vs. Durban	−0.105	0.109	−0.962	0.773
**Female: Cape Town vs. Mauritius**	**0.445**	**0.108**	**4.124**	**0.007**
Female: Cape Town vs. Réunion	0.192	0.113	1.969	0.366
**Female: Durban vs. Mauritius**	**0.550**	**0.060**	**9.183**	**<0.001**
**Female: Durban vs. Réunion**	**0.297**	**0.069**	**4.299**	**<0.003**
**Female: Mauritius vs. Réunion**	**−0.253**	**0.067**	**−3.78**	**0.009**
Male: Cape Town vs. Durban	−0.057	0.110	−0.516	0.954
**Male: Cape Town vs. Mauritius**	**0.391**	**0.108**	**3.608**	**0.016**
Male: Cape Town vs. Réunion	0.136	0.113	1.203	0.636
**Male: Durban vs. Mauritius**	**0.447**	**0.061**	**7.279**	**<0.001**
Male: Durban vs. Réunion	0.193	0.070	2.756	0.062
**Male: Mauritius vs. Réunion**	**−0.254**	**0.068**	**−3.747**	**0.009**

**(C) Multiple comparisons between sexes**				

Contrasts	*β*	*SE*	*t*	*p*_*adj*_
**Cape Town: Females vs. Males**	**0.108**	**0.030**	**3.565**	**<0.001**
**Durban: Females vs. Males**	**0.157**	**0.022**	**6.922**	**<0.001**
**Mauritius: Females vs. Males**	**0.054**	**0.022**	**2.507**	**0.013**
**Réunion: Females vs. Males**	**0.053**	**0.026**	**2.067**	**0.039**

(A) The table gives coefficient estimates (*β*) that are representative of log_10_-transformed summed parotoid gland area with their corresponding standard errors (*SE*) and *t* values. Variance estimates (*σ*^*2*^) are supplied for residuals and random effects. All significant values (*p* < 0.05) are bolded. Reference levels for each categorical variable are supplied in parentheses following the variable name. (B) Post-hoc multiple comparisons of absolute parotoid gland size between toads of the same sex and different locations. (C) Post-hoc multiple comparisons investigating sexual absolute parotoid gland size dimorphisms in toads of the same location. In multiple comparisons, *p* values were Tukey-adjusted. All significant values (*p* < 0.05) are bolded.

### Composition of the toxin compounds in the parotoid gland secretion

**Figure 3:**
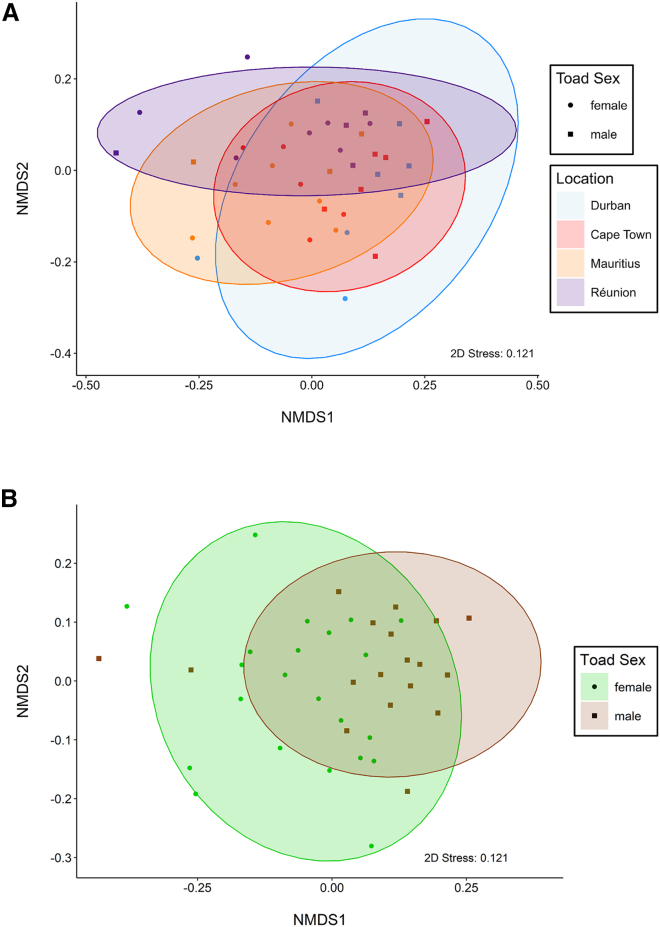
Non-metric multi-dimensional scaling-plots (NMDS-plots) showing location- and sex-specific differences in toxin composition (A) NMDS-plot showing differences in compound composition of the parotoid gland secretion of toads from different locations (Durban—blue, Cape Town—red, Mauritius—orange, and Réunion—violet). (B) NMDS-plot showing differences in compound composition of the parotoid gland secretion of female (green circle) and male (brown square) toads.

Overall, 15 different toxin compounds were tentatively identified in the samples using liquid chromatography high-resolution mass spectrometric (LC-HR-MS) analysis. All of these compounds are derivates of bufadienolides, known for their cardiotoxic effects^34^^,^^35^^,^^55^^,^^56^^,^^57^ and were present in almost all samples. In two samples, only 14 of the 15 compounds were detected. However, the missing compound made up only 0.3% ± 0.1% (mean ± SE henceforth) of the relative amount of the toxin compounds in a sample (Table S3). Thus, the richness of the toxic substances in the samples was highly similar. However, most compounds were present in only very low proportions in the samples (eleven compounds had average proportions of lower than 5%), while one compound made up 45.1% ± 0.9% of the toxin compounds and three other compounds had proportions of 14.3% ± 1.0%, 13.5% ± 0.5%, and 11.1% ± 0.7% of the overall relative amount of toxins (Table S3, Figure S1). Therefore, this high variation in the average proportions of the compounds, created the possibility of strong differences in evenness (i.e., Shannon diversity) among groups of samples (i.e., locations and sexes). Males had a lower evenness of toxin compounds than females, and toads from Réunion had a higher evenness of toxin compounds than toads from Mauritius and Cape Town (Table S4, Figure S2). No other differences regarding the analysis of compound evenness were significant. Similarly, there were no differences in the dispersion of compounds between locations (*p* = 0.845, *F* = 0.272) or sexes (*p* = 0.463, *F* = 0.549). However, the composition of compounds (i.e., the centroid location) differed by location (*p* = 0.007, *R*^*2*^ = 0.163, Figure 3A) and by sex (*p* = 0.002, *R*^*2*^ = 0.106, Figure 3B). Specifically, compound composition differed between Durban and Cape Town (*p* = 0.006, *R*^*2*^ = 0.302), Durban and Mauritius (*p* = 0.009, *R*^*2*^ = 0.322), Durban and Réunion (*p* = 0.017, *R*^*2*^ = 0.297), Cape Town and Mauritius (*p* = 0.020, *R*^*2*^ = 0.263), Cape Town and Réunion (*p* = 0.004, *R*^*2*^ = 0.288) but not between Mauritius and Réunion (*p* = 0.139, *R*^*2*^ = 0.161).

Results from the generalized linear latent variable model indicated many significant differences in proportions between compounds (Tables S3 and S5) and multiple strong correlations between specific compounds (Figure S3). Five compounds differed in proportions between locations (Table S6). However, all five compounds occurred in only very low proportions in the samples (average proportions <3%, Table S3). In contrast to the overall composition that differed between Durban and Cape Town, no comparisons of specific compound proportions were significantly different between Durban and Cape Town (Table S6). Four compounds showed sex-specific differences in proportions (Table S7). Two of these compounds occurred in high proportions in the samples (45.1% ± 0.9% and 13.5% ± 0.5% mean proportion ±SE, respectively; also see Table S3).

### Potential toad predators in each location

**Figure 4:**
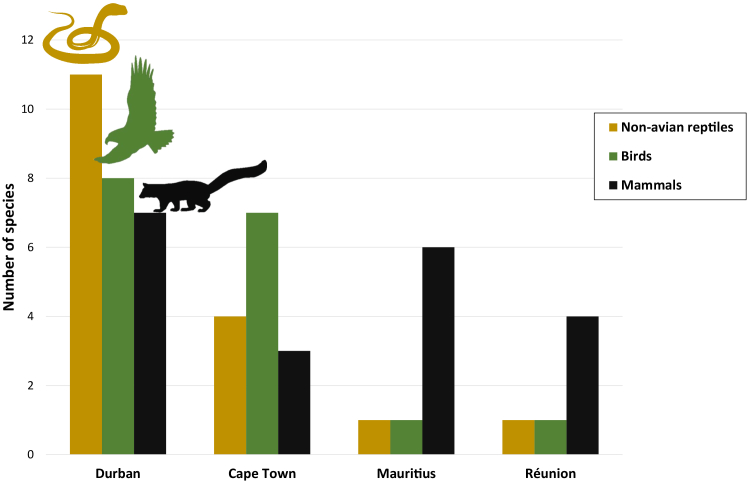
Number of potential predator species by location Each bar shows the total number of potential predator species for Guttural Toads in a given location. The colors correspond with the type of predator (gold—non-avian reptiles, green—birds, and black—mammals).

Overall, 26, 13, 8, and 6 potential Guttural Toad predator species were identified for Durban, Cape Town, Mauritius, and Réunion, respectively (Figure 4, Table S8).^43^^,^^58^^,^^59^^,^^60^^,^^61^^,^^62^^,^^63^^,^^64^^,^^65^^,^^66^^,^^67^^,^^68^^,^^69^^,^^70^^,^^71^^,^^72^^,^^73^^,^^74^ Notably, the number of non-avian reptile and bird predators is much lower in Mauritius and Réunion (*n* = 2 on both islands, respectively) compared to Durban (*n* = 19) while in Cape Town, 11 non-avian reptile and bird predator species were identified (Figure 4, Table S8). In Mauritius and Réunion all identified potential vertebrate predators are introduced species (Table S8).

## Discussion

### The role of enemy release in the changes of Guttural Toad chemical defenses

Since their introduction to Mauritius and Réunion approximately 100 years ago, the chemical defenses of Guttural Toads have changed—quantitatively as the parotoid glands show marked decreases in size and qualitatively as the overall compound composition and the proportions of specific compounds have changed—when compared to the population of origin in Durban. Approximately 25 years after their introduction to Cape Town, the Guttural Toads show changes in their toxin composition but do not show changes in their parotoid gland size nor proportions of specific toxins compared to toads of the population of origin in Durban. These two independent invasion events that originated from the same population highlight potential causes and constraints of the changing chemical defenses of a successful invasive toad species.^46^ Consistent with the enemy release hypothesis, the introduced toads are confronted with fewer potential predator species in each of their non-native ranges. While the number of potential predator species halved in Cape Town, on Mauritius and Réunion, where no native anuran communities exist,^53^ the number of potential predators for Guttural Toads is even lower. Therefore, the abundance and diversity of predators is likely much lower on Mauritius and Réunion and the toads there show reduced investment into parotoid gland tissue and changes in their toxin profile. These results are in line with two previous studies that investigated changes in morphology and escape performance in Guttural Toads on Mauritius and Réunion compared to toads from Durban.^50^^,^^52^ The results indicated reduced body size, limb length, escape speed, and endurance capacity in the invasive island toads. As these traits together with the chemical defenses investigated in this study are highly relevant in a predator escape context, it is likely that the changes we are observing are the result of reduced predation pressure. On the other hand, in Cape Town, where a rich community of anurans exists, including the closely related Western Leopard Toad (*Sclerophrys pantherina*), the predation pressure experienced by Guttural Toads is likely higher compared to Mauritius and Réunion and we have only observed changes in the general composition of the toxin secretion.

While our results, taken together with the results of Baxter-Gilbert et al. (2020)^50^ and Baxter-Gilbert et al. (2022),^52^ provide compelling evidence for enemy release as the driver of changes in the invasive toads, they are correlative in nature and thus, should be supplemented by future studies to provide further evidence. A common-garden-experiment with toads from all four populations raised from eggs should be conducted to investigate the role of environmental and genetic causes behind the apparent reduction in defensive traits that we have reported. The size of the parotoid glands and the composition of the secretion are known to be plastic as well as to show heritable differences in other toad species.^5^^,^^6^^,^^23^^,^^26^^,^^27^^,^^75^^,^^76^^,^^77^^,^^78^^,^^79^^,^^80^^,^^81^ The toads should be followed until adulthood to investigate differences in parotoid gland size and the composition of toxic secretion. If differences arise, these should be the result of heritable effects. Mating trials with subsequent counting of the number of hatched tadpoles could be conducted to investigate any trade-offs between chemical defenses and reproductive output. Lastly, toads that originated from the four different populations should be exposed to different predators (e.g., a snake such as *Limaformosa capensis*, a bird such as *Lanius collaris*, and a mammal such as *Rattus norvegicus*) to evaluate the adaptive nature of the chemical defenses of Guttural Toads when faced with different predators (see also^82^). These experiments would provide a better understanding of the mechanism of change (i.e., phenotypic plasticity vs. heritable changes) and the adaptive value of the changes (i.e., improved fitness due to resource allocation into reproductive traits at the cost of decreased survival under predation threat).

Other drivers of the changes in chemical defenses should, however, not be disregarded. While the bufadienolides of Guttural Toads like those of other bufonids are biosynthesized and not directly sequestered from the diet,^83^^,^^84^ differences in prey availability across the different ranges^85^ might influence the ability of the toads to grow parotoid gland tissue and synthesize specific compounds due to limitations posed by specific nutrients (but see^86^). Similarly, other bioactive compounds available in the environment can affect the chemical defenses of toads.^78^ Another important function of toad chemical defenses is mediating competition. For example, tadpoles of the Common Toad (*Bufo bufo*), synthesize higher amounts of bufadienolides under higher levels of competition^76^^,^^77^ as these enhanced chemical defenses can mitigate risks posed by higher levels of competition such as cannibalism or disease transmission. Furthermore, Mayer et al. (2021) showed that the skin secretions of Cane Toads can reduce the infection success and longevity of lungworm parasites.^87^ While there is no data available on differences in density among the populations of Guttural Toads, these toads have been observed in high numbers in each of the four ranges studied here (personal observations). The possibility of enemy release from parasites in the invasive populations is still very conceivable, especially given that Mauritius and Réunion have no native anuran fauna^53^ and therefore, might not harbor any parasite species that are capable of infecting toads. Research has found that the Cape Town Guttural Toad population showed lower diversity of six parasite species compared to toads from Durban (although this difference was statistically non-significant)^88^ and could therefore benefit from an escape from parasites.

Another potential cause of the differences in chemical defenses could have been genetic bottlenecking during the introduction of the toads to the new ranges.^89^ In line with a deliberate release of likely a considerable number of Guttural Toads on Mauritius, the genetic diversity of the Mauritius population is only slightly lower than the diversity of the source population.^46^ The genetic diversity then further decreased when a sub-population from Mauritius was introduced to Réunion five years later.^46^ However, in Cape Town where the colonization was likely inadvertent, the genetic diversity is the lowest compared to the founder population.^46^ Therefore, if the patterns that we observed in this study were solely a result of reduced genetic diversity due to a genetic bottleneck during the introduction, we would expect the strongest changes in the Cape Town population and the weakest changes in the Mauritius population. However, rather the opposite is the case. While genetic bottlenecking might have played a role in the drastic changes in the chemical defenses of the Guttural Toad, it is likely not the main cause.

Instead, the time span since colonization might play a more important role as the separation from the source population for Mauritius and Réunion occurred 100 years ago (approx. 50 generations), while Cape Town was invaded roughly 22 years ago (approx. 11 generations) when the samples were taken. Given that founder effects for the introduction to Mauritius and subsequently to Réunion are unlikely,^46^ but selection had almost five times more opportunity to act on the chemical defenses of the toads in Mauritius and Réunion compared to Cape Town, it is possible that in the future the chemical defenses of the Cape Town population might further diverge from the source population (if the changes we observed here represent heritable differences^49^^,^^90^). Indeed, previous research on the temporal aspects of adaptations in the defensive traits of anurans have shown that the time span since introduction can have an important effect on the type and strength of the defenses.^91^^,^^92^^,^^93^^,^^94^ Given the considerably higher variance in relative parotoid gland size in Cape Town compared to the other population (see Figure 2), it is possible that this trait is currently changing. Considering that Guttural Toads have a lifespan of five to eight years,^95^ it is very possible that among the sampled toads from Cape Town, several generations were sampled which might have resulted in the high variance in relative parotoid gland size observed here. Given an extirpation program is continuing in Cape Town,^96^ specimens should continuously be available for longitudinal studies to observe changes in for example parotoid gland size that could reveal the potential of adaptive evolution before our eyes.

### The significance of changing toxin compositions

The changes in toxin composition in all invasive populations as well as changes in the proportions of specific compounds in Mauritius and Réunion toads compared to the founder population in Durban indicate that the toad’s chemical defenses do not only change in quantity (i.e., parotoid gland size) but also in quality. Despite our low sample size for the analysis regarding the toxin compounds, we have found changes in the overall composition of the toxic secretion as well as the proportions of specific compounds while no compound was lost or gained. However, it is not unlikely that more minute differences might have been missed due to the limited statistical power of our analyses caused by our low sample size. Therefore, studies with more robust statistical analyses are needed to supplement our findings. Unfortunately, at this time, there is not enough information available on the anti-predator function and metabolic pathways of specific bufadienolides (but see^55^^,^^56^^,^^57^) to draw any conclusions of the ecological and evolutionary relevance of the changes that we have observed (see also^97^). However, given the lower number of potential predators in the invasive ranges, we could speculate that any changes in specific compounds could indicate that these compounds are energetically cheaper to produce and less-efficient against specific predators (e.g., snake predators, see Table S8). Furthermore, all compounds that showed changes in the invasive island populations, made up only very low proportions of the toxic cocktails of the toads (see Tables S3 and S6). Therefore, relaxed selection could act on these compounds first.^98^ Interestingly, no compound completely disappeared in the chemical cocktail of the invasive populations. Given the drastic reductions in parotoid gland size observed in the invasive island populations and the comparatively weaker changes in toxin composition, it appears that the metabolic pathways to produce the specific bufadienolides are more fixed compared to reductions in size of the parotoid gland tissue.

Comparing sexes, we see a different picture. Two of the most abundant compounds show higher proportions in male secretions compared to female secretions (see Tables S3 and S7). Therefore, these compounds could be involved in male-male conflict^99^^,^^100^^,^^101^ or could reflect sex-specific differences in predation risk (e.g., because male toads emit advertisement calls, while females are silent, males might be more susceptible to predators that use auditory and visual cues^102^). Similarly, females showed greater evenness in toxin composition than males (see Figure S2). This might be related to the increased proportions of two highly abundant compounds in males but does not necessarily indicate that females possess more potent chemical defenses than males as the identity and interaction of specific compounds in the secretion are likely more important.

Previous studies that investigated changes in toxin composition between native/invasive or rural/urban populations of toads focused on either specific compounds or a sub-class of bufadienolides^5^^,^^26^ with the justification that these compounds or sub-classes of bufadienolides are the most potent compound in the cocktail of toxins. However, as suitable bioassays on the potency of bufadienolides have not been conducted^55^^,^^56^^,^^93^ and the secretions of invasive toads consist of many bufadienolide compounds (e.g., in Cane Toads:)^27^^,^^101^, possible variation in toxin composition among the invasive populations, cannot be fully investigated by focusing on only one or a few compounds. With further knowledge advanced by analytical tools and ecologically relevant bioassays, future studies will be able to unveil the relevance of changes in toxin composition and the significance of specific compounds in the cocktail of bufadienolides.

### Why relative and absolute parotoid gland size matter

In our study, we investigated changes in relative parotoid gland size as well as in absolute parotoid gland size in contrast to most other studies that focused on relative parotoid gland size (but see^38^^,^^39^). Studying changes in parotoid gland size in relation to body size is interesting as it provides an insight into the relative investment of a toad in its chemical defenses.^103^^,^^104^^,^^105^^,^^106^ Therefore, it is especially relevant from an evolutionary perspective where knowledge of changes in the investment into a defense is related to changes in the environment is of interest. On the other hand, when we are interested in the ecological impact of an invasive toad, the absolute gland size as a proxy for volume of secretion might be more important as it is the absolute volume of toxin a predator ingests and not the volume of toxin in relation to the body size of the prey that ultimately would affect the predator.^107^ Therefore, a predator ingesting a toad on Mauritius or Réunion will be faced with an even lower dose of toxins during ingestion (see results) compared to a predator ingesting a toad in Durban. While it has already been shown that the toads show phenotypic changes in accordance with the novel selective environment,^47^^,^^48^^,^^50^^,^^52^^,^^108^^,^^109^ their chemical defenses have largely been overlooked so far. Here, we show that the toads not only change their chemical defenses in accordance with the environment but also that these changes can have important ecological consequences.

### Limitations of the study

Our study entails some notable limitations. First, the mechanism causing the changes in the invasive populations remains unknown as our study was correlative and we were unable to distinguish between the effects of phenotypic plasticity and evolution. Second, the biological significance of the changes in toxin composition and of specific toxic compounds remains unresolved as we are currently missing meaningful bioassays important information about metabolic pathways. Third, our low sample size in the chemical analysis of the parotoid secretions might have limited our ability to identify more minute differences in toxin composition or proportions.

## Resource availability

### Lead contact

Requests for further information and resources should be directed to and will be fulfilled by the lead contact, Max Mühlenhaupt (max.muehlenhaupt@uni-bielefeld.de).

### Materials availability

This study did not generate new unique reagents.

### Data and code availability

• All data reported in this study including the mass spectrometry data generated during the analysis of the parotoid gland secretion have been deposited at OSF (Open Science Framework) and are publicly available as of the date of publication at https://osf.io/c6apy/overview?view_only=e2b68a71c90c452284f529cc1e649ddd.
• All original code has been deposited at OSF (Open Science Framework) and is publicly available at https://osf.io/c6apy/overview?view_only=e2b68a71c90c452284f529cc1e649ddd as of the date of publication.

## Acknowledgments

M.M. received funding by the 10.13039/501100001659German Research Foundation (DFG) as part of the CRC TRR 212 (NC^3^)—Project number 316099922 and is a member of project A04—Project number 396777092. We would like to thank M.M.’s colleagues at the Animal Behavior Building in Bielefeld and participants of the World Congress of Herpetology 2024 for helpful feedback on this project. Further, we would like to thank the Botanical Garden of Durban for permission to conduct fieldwork at night. We are grateful for the valuable feedback of three anonymous reviewers and the editors of iScience that helped strengthen this manuscript. In memory of Dr. G. John Measey: John, this one is for you!

## Author contributions

M.M. contributed to study conception, data acquisition and analysis, writing (original draft and review), visualization and project administration. J.B.-G. contributed to study conception, data acquisition, writing (review) and project administration. J.L.R. contributed to data acquisition and analysis and writing (review). B.G.M. contributed to data acquisition, writing (review) and project administration. N.S.D., C.B., F.B.V.F., and X.P. contributed to data acquisition and writing (review). A.d.V. and W.A.L.v.O. contributed to data analysis, visualization and writing (review). J.M. contributed to study conception, data acquisition and analysis, writing (original draft, review), visualization, project administration and funding acquisition.

## Declaration of interests

The authors declare no competing interests.

## STAR★Methods

### Key resources table

**Table:** 

REAGENT or RESOURCE	SOURCE	IDENTIFIER
**Deposited data**		

Mass spectrometry data	this paper	https://osf.io/c6apy/overview?view_only=e2b68a71c90c452284f529cc1e649ddd
Gland size and toxin composition data	this paper	https://osf.io/c6apy/overview?view_only=e2b68a71c90c452284f529cc1e649ddd

**Software and algorithms**		

RStudio	https://posit.co/downloads/	v. 4.4.0
ImageJ	https://imagej.net/ij/download.html	v. 1.54
Map of life	https://mol.org/	N/A

### Experimental model and study participant details

Collection and sampling of toads were carried out in accordance with the ethical guidelines of Stellenbosch University Research Ethics Committee (ACU-2019-13154 & ACU-2020-10386) and University of KwaZulu-Natal Animal Research Ethics Committee (AREC/009/020) and the South Africa National Standard: The Care and Use of Animals for Scientific Purpose (SANS 10386:2008). All methods including euthanasia of invasive specimen were approved by Ezemvelo KZN Wildlife Ordinary Permit (OP 4072/2019), Cape Nature Permit to Export and Transport Protected Wild Animals (CN13-59-12357, CN44-8716662 & CN44-87-15961) and Mauritian National Parks and Conservation Services (NP 46/3 V3). After sampling, toads were either humanely euthanized or released at their site of capture, depending on the protocols and requirements of our scientific permits and animal ethics approvals. The sample sizes for each measurement, location, and sex are reported in the “method details” section.

### Method details

#### Study system

Guttural Toads are large bufonids (snout-vent length (SVL) up to 140 mm)^110^ with a broad distribution in sub-Saharan Africa (Figure 1A). Like other bufonids, adults of the species have two prominent parotoid macroglands that store and produce toxin compounds,^111^ which are exuded from the glands when the toad is attacked (Figure 1B). Like in other toads, these compounds have cardiotoxic and nauseating effects upon ingestion,^111^^,^^112^ and thereby, often repel or even kill predators.^10^^,^^113^

Guttural Toads have three known invasive populations on Mauritius and Réunion, and in a peri-urban area of Cape Town. All three invasive populations have originated from the same clade located in the area of Durban, South Africa.^46^ Adult toads were introduced to Mauritius in 1922, and from Mauritius to Réunion in 1927; both deliberate attempts for bio-control of invertebrate pests.^46^^,^^53^ Conversely, their introduction to Cape Town in 2000 likely occurred accidentally via a consignment of aquatic plants containing eggs and larvae.^45^^,^^46^ Mauritius and Réunion are similarly sized islands (2040 km^2^ and 2512 km^2^, respectively) with tropical climates,^114^ while Cape Town represents a mainland habitat with a much more mediterranean climate.^48^ Thus, the invasive populations are experiencing different selection regimes and vary in the duration of exposure to site-specific selection (approximately 51, 49 and 12 generations for Mauritius, Réunion and Cape Town, respectively; Figure 1A).^45^

#### Parotoid gland size measurements

Between late 2018 and early 2023, adult Guttural Toads were collected from six different sites in the area of Durban (*n*_Females_ = 89, *n*_Males_ = 71), from the peri-urban area of Cape Town (*n*_Females_ = 45, *n*_Males_ = 45), from ten sites on Mauritius (*n*_Females_ = 99, *n*_Males_ = 79), and from four sites on Réunion (*n*_Females_ = 71, *n*_Males_ = 76). Because of low sample sizes (lower than four samples per site), three sites on Mauritius were removed from the analysis (the sample sizes above represent the final number of samples per location, see Table S1 for an exhaustive summary of sample sizes).

To measure the size of the parotoid glands of each toad, a ruler or piece of grid paper was placed next to the parotoid gland on each side of the toad’s head. A photo was taken for each parotoid gland and, using the size standard in the photo, the area of the parotoid gland was measured in ImageJ by outlining the shape of the gland and using the *measure* function.^115^ In addition, each toad’s snout-vent-length (SVL) was taken by placing a ruler on the ventral side of the toad and measuring the shortest distance from the snout tip to the cloaca. Only sexually mature toads were considered in the analysis. Given already known pronounced differences in the body size comparing toads from Durban with toads from Mauritius and Réunion,^50^^,^^52^ we used different size thresholds to determine sexual maturity for each location. For Mauritius and Réunion, we applied the same size threshold as^50^^,^^52^: 38 and 36 mm SVL, respectively. For Durban, we used 48 mm as the size threshold because this was the shortest SVL of a male displaying the yellow throat patch that was captured for another experiment.^49^^,^^90^ This yellow throat patch is indicative of sexual maturity of males for the species and is used to discern males from females as females do not display this throat patch.^110^ Similarly, for Cape Town we used 49 mm SVL as the size threshold separating adult males from juveniles.

#### Sampling of the parotoid secretion

For a subset of toads (Durban: *n*_Female_ = 3, *n*_Male_ = 5; Mauritius: *n*_Female_ = 7, *n*_Male_ = 3; Réunion: *n*_Female_ = 7, *n*_Male_ = 4; Cape Town: *n*_Female_ = 5, *n*_Male_ = 6), we sampled the parotoid gland secretion (Table S1). Using nitrile gloves, we gently squeezed the toad’s left parotid gland between thumb and first finger using light pressure so that toxins were emitted, often projected from, the parotoid gland. Simultaneously, we held a sterile cotton swab close to the gland so that the swab was coated with toxin. Toads were returned after sampling without any harm to the animal. The cotton swabs were stored at -20°C before they were transferred to -80°C storage within two weeks of sampling. Thereafter, the secretion samples were shipped to the Department of Chemistry and Polymer Science of Stellenbosch University in South Africa for chemical analysis.

#### Chemical analysis of the parotoid secretion

The collected swabs were desorbed in Eppendorf tubes using 1 mL methanol under sonication for 5 min, before being centrifuged at 14000 rpm for 5 min. Extracts were analyzed on an Acquity UPLC instrument coupled to a Synapt G2 quadrupole-time-of-flight (Q-TOF) mass spectrometer (Waters, Milford, MA) equipped with an Acquity BEH C18 column (2.1 mm × 100 mm, 1.7 μm, Waters) using an injection volume of 2 μL. The mobile phases consisted of 0.1% formic acid in Milli-Q water (solvent A) and acetonitrile (solvent B), at a flow rate of 0.4 mL/min. The initial conditions were 0% B, maintained for 0.5 min, followed by a linear increase to 100% B at 12 min, kept for 0.5 min before returning to the initial conditions at 13 min. The column was re-equilibrated for 2 min at 0% B (total run time 15 min). The column temperature was maintained at 50 °C. Detection was performed using photodiode array (PDA) detection between 230 and 500 nm (20 Hz) and positive electrospray ionisation (ESI^+^) MS. The following ionisation parameters were used: capillary voltage: 2.5 kV; cone voltage: 15 V; source temperature: 120 °C; cone gas flow (N_2_): 50 L/h; desolvation gas flow (N_2_): 650 L/h; desolvation temperature: 275 °C. Both low (4 eV) and high (ramped from 10 to 30 eV) collision energy data were recorded by employing MS^E^ mode. Data were acquired from 120 to 1500 amu (low collision energy) and 40 to 1500 amu (high collision energy) using a scan time of 0.2 s (5 Hz). Sodium formate was used for the calibration of the instrument and leucine enkephalin was used as the lock mass calibrant (m/z 556.2771, [M+H]^+^). Toxins were tentatively identified based on careful analysis of relative chromatographic retention and low- and high collision energy HR-MS data, which provided the molecular formula and fragmentation information, respectively, compared to literature.^35^^,^^55^^,^^56^^,^^57^

#### Literature search on potential toad predator species in each location

In order to estimate the predator pressure a Guttural Toad receives in the invasive ranges and in the origin location of the invasive populations (Durban), we conducted a search of the scientific literature and of field guides that report predation of a certain species on adult Guttural Toads or closely related toad species. We then confirmed the existence of that species in a study location using biodiversity datasets available in “Map of Life” (mol.org). We classified potential predator species by taxonomic groups (non-avian reptiles, birds, mammals) as predation tactics and susceptibility to toad venom might differ by taxonomic groups.^116^^,^^117^^,^^118^^,^^119^ We only considered vertebrate predators as the data on invertebrate predators of Guttural Toads is sparse and we were not able to track down invertebrate predators to the species level. Furthermore, many invertebrate predators might show immunity to toad venom, thereby not acting as selective agents on the chemical defenses of Guttural Toads.^120^^,^^121^ The literature search was first conducted in October 2021 and another search was conducted as an update to the initial search in January 2025.

### Quantification and statistical analysis

All statistical analyses were carried out in R version 4.4.0.^122^ Prior to any analysis, we explored our data according to.^123^ Following, model fitting, we checked if the models met the model assumptions using the function *simulateResiduals* in the package “DHARMa”^124^ and *check_model* in the package “performance”.^125^ To conduct Tukey-adjusted multiple comparisons between locations and sexes, we used the function *emmeans* in the R package “emmeans”.^54^ To analyze differences in parotoid gland size of Guttural Toads, we used linear mixed effects models (LMMs) using the function *lmer* in the R package “lme4”.^126^ We summed the area of the left and right parotoid gland per toad as the amount of toxin produced is related to the overall size of the tissue.^112^ Parotoid gland area and SVL were log_10_-transformed to ensure linear relationships between our model variables.^127^ In an initial analysis, we tested for differences between urbanized and rural sites as urbanization has been shown to be an important factor influencing the chemical defenses of other toad species.^5^^,^^6^^,^^81^ Sites were assigned urban or not based on established criteria (Table S1).^49^^,^^52^^,^^90^^,^^128^ We used a LMM with the summed and log_10_-transformed gland area as dependent variable and fixed effects of location, sex, urbanization and log_10_-SVL as well as site as a random intercept that indicated no differences in parotoid gland size between urban and rural sites in the same location (*p* = 0.95, Table S2). Therefore, we did not consider urbanization for any other subsequent models.

To test differences between locations and sexes in parotoid gland size, we used LMMs with the summed and log_10_-transformed gland area as dependent variable and location, sex and their interaction as explanatory variables. As a random intercept, we included the identity of the site the toad was collected in to avoid pseudo-replication in case toads from the same site are more related to each other than toads from different sites. For one model, we included log_10_-SVL as a covariate in order to investigate differences in parotoid gland area in relation to body size (i.e., relative parotoid gland area). For the other model, we did not include log_10_-SVL to examine differences in the absolute parotoid gland area. We calculated differences in relative parotoid gland area between invasive toads and toads from Durban based on back-transformed estimated marginal means provided by *emmeans*.^54^ We calculated differences in absolute parotoid gland area between invasive toads and toads from Durban based on back-transformed averages of the raw data for each group.

To analyze differences in the composition of the toxin compounds found within the parotoid secretion, we used relative amounts (i.e., toxin-specific peak area obtained from integrated extraction ion chromatograms (EICs), divided by the total EIC peak areas of all toxins) because the amount of secretion collected was not standardized during the sampling.^129^^,^^130^ To compare the α-diversity of the toxin compounds between locations and sexes, we calculated the sum number of substances (i.e., richness) and the Shannon diversity index for each sample using the *diversity* function in the package “vegan”.^131^ Shannon diversity was subsequently used as the dependent variable in a linear model^122^ with location and sex as explanatory variables. Given low sample sizes (see above) and that usually all samples from one location were taken at one site (Table S1), we did not consider site or an interaction of sex and location in any analysis investigating differences in the composition of the toxin compounds of the parotoid secretion. To test for location- and sex-specific differences in the overall composition (i.e., β-diversity) of the toxin compounds, we used a permutational multivariate analysis of variance (PERMANOVA) on Bray-Curtis-dissimilarities supplied via the function *adonis2* in the “vegan” R package^131^ with location and sex as independent effects. The PERMANOVA was conducted with 9,999 permutations and the marginal effect of each term was tested. Because the first PERMANOVA indicated differences by location (see results), we ran pairwise PERMANOVAs using the *pairwise.adonis2* function with 999 permutations in the “pairwiseAdonis” R package.^132^ We included location and sex as independent effects and used Benjamini-Hochberg-correction for *p*-value adjustment.^133^ Subsequent to the PERMANOVAs, we tested for heterogeneity of variances between locations and sexes using *vegdist* and *betadisper* in “vegan”^131^ to test whether potential effects identified by the PERMANOVA are due to differences in the location of the centroids (i.e., groups differ in composition) or due to differences in dispersion of the groups (i.e., groups differ in the variance of composition).^134^ To visualize differences in composition, we used Non-metric Multi-Dimensional Scaling (NMDS) with *metaMDS* in “vegan”,^131^ considering two to four dimensions for the NMDS. We chose two dimensions for the final NMDS as the stress value was still acceptable (0.121) and enabled us to plot all dimensions in a 2-D-plot.

To analyze differences in the relative abundance of specific compounds in the secretion, we used a generalized linear latent variable model (GLLVM) with the function *glmmTMB* provided by the R package “glmmTMB”.^135^ This model included a reduced-rank variance-covariance structure with two rank parameters by including a random slope of compound across samples (i.e., toad IDs) to enable the analysis of multivariate abundance data.^136^ As the dependent variables were the proportions of the toxin compounds, we used a beta distribution with logit-link function in the GLLVM. As fixed effects, the model included the ID of the toxin compound (to control for differences between compounds) as well as location and sex of the toad sampled. Therefore, we were able to test for location- and sex-specific differences of the relative abundance of compounds while controlling for the correlation among compounds and obtained a correlation matrix of the toxin compounds while controlling for sex- and location-specific effects. To conduct multiple comparisons between compounds, and between locations and sexes for specific compounds, we used the function *emmeans* with Tukey-adjusted *p*-values.^54^
