# Supplementary material for: Changes in the chemical defenses of an invasive toad indicate drivers and limitations of adaptation
Source: iScience. 2026 Mar 25;29(4):115401. doi: 10.1016/j.isci.2026.115401 (PMC13091458; doi:10.1016/j.isci.2026.115401)
Supplement: Document S1. Figures S1–S3 and Tables S1–S8 [file mmc1.pdf]

iScience, Volume *29*

Supplemental information

Changes in the chemical defenses of an invasive toad indicate drivers and limitations of adaptation

Max Mühlenhaupt, James Baxter-Gilbert, Julia L. Riley, Buyisile G. Makhubo, Nhlanhla S. Dludla, Cláudia Baider, F.B. Vincent Florens, Xavier Porcel, André de Villiers, Willem A.L. van Otterlo, and John Measey

Supplemental figures

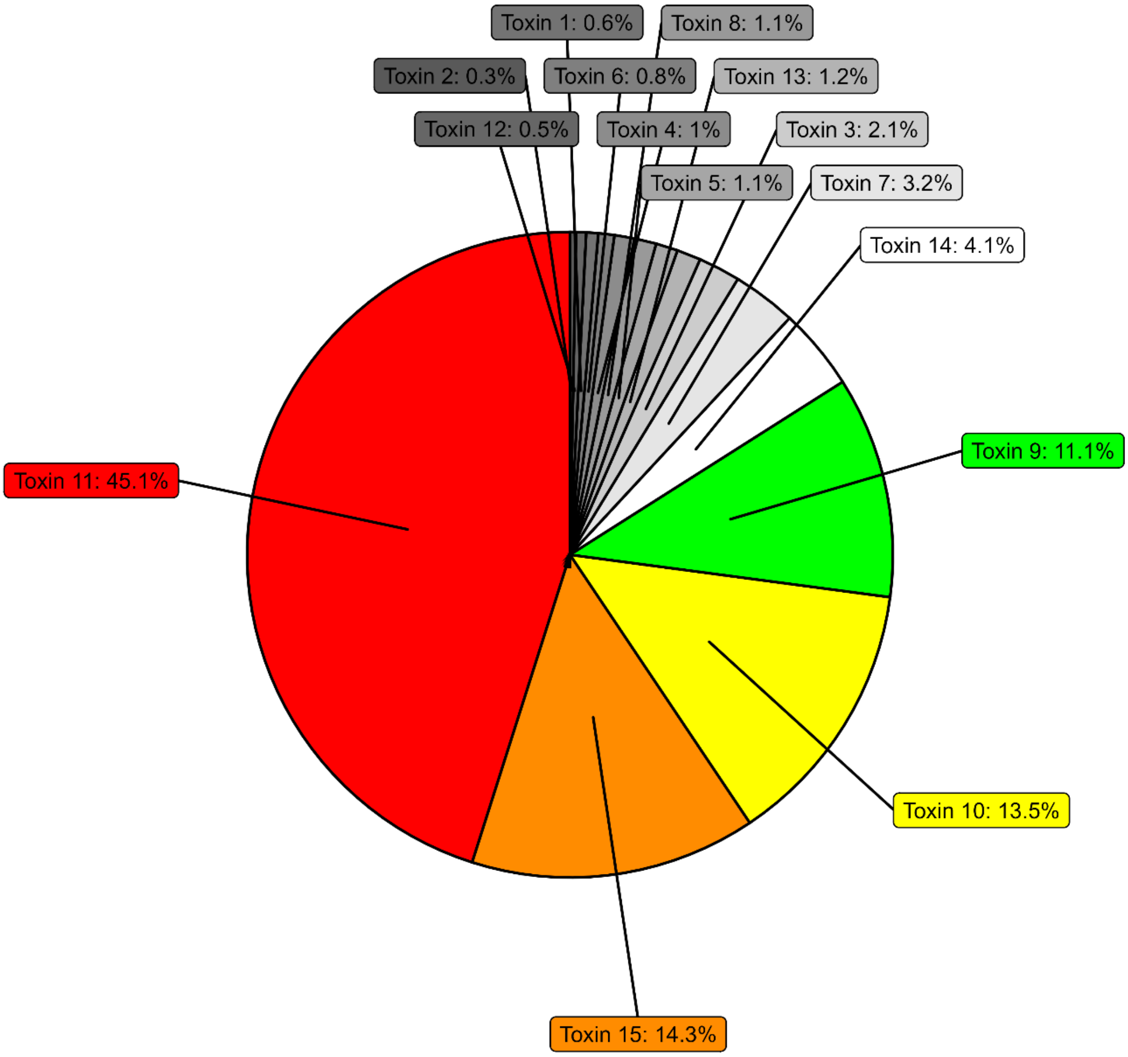

Fig S1. Average proportions of each identified toxin compound. This pie chart shows the average proportions (in %) of each bufadienolide compound identified in the parotoid gland secretion samples.

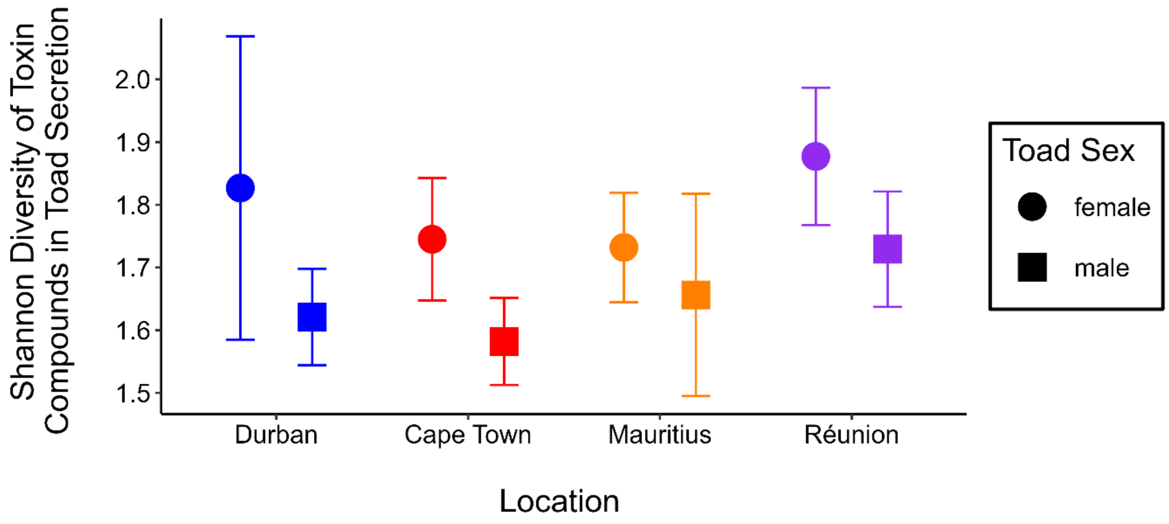

Fig S2. Compound evenness (Shannon diversity) among locations and sexes. Presented are the Shannon diversity indices of females (circles) and males (squares) from each studied location (Durban – blue, Cape Town – red, Mauritius – orange, Réunion – violet). Presented are means with their corresponding 95 % confidence intervals (using the *t*-distribution with *n*-1 degrees of freedom).

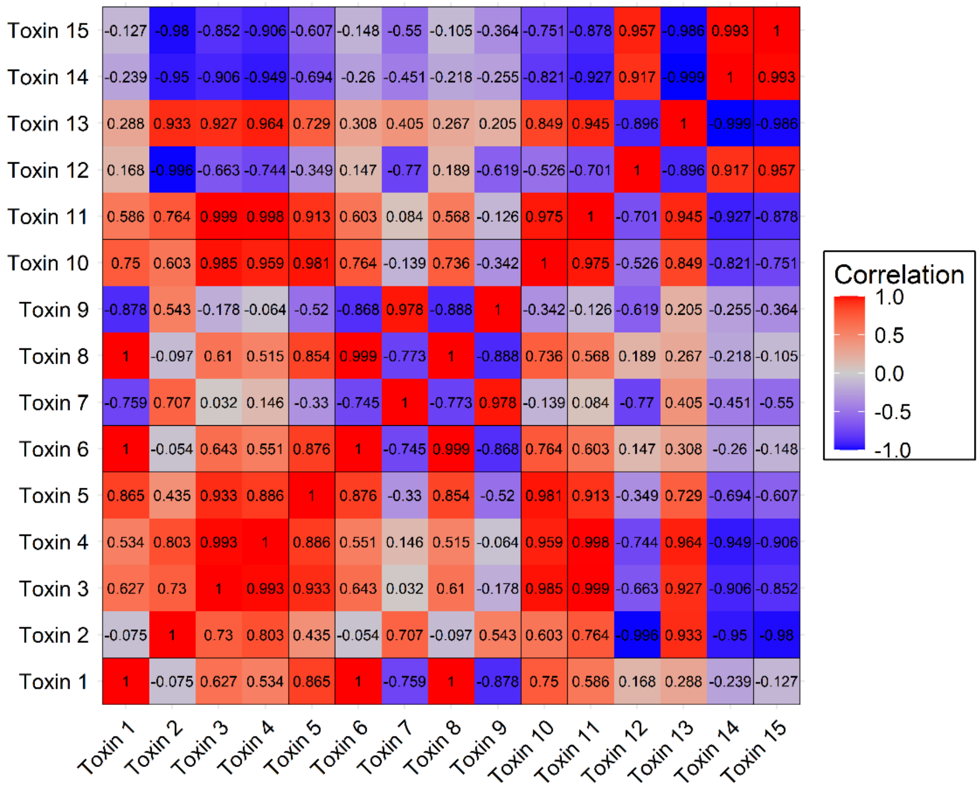

Fig S3. Correlation plot of compounds. Presented are the covariance estimates extracted from the variance-covariance-matrix of the generalized linear latent variable model investigating differences in compound proportions. The covariance estimates displayed here are standardized for location- and sex-specific differences.

Supplemental tables

Table S1. Overview of sample sizes and sampling sites. This table shows the sample sizes (*n*) by sex for each site (including GPS coordinates) belonging to one of the four locations (Durban, Mauritius, Réunion, and Cape Town) where toads were sampled. Additionally, we include whether the site was considered urbanized or not as we initially tested for differences between toads from urbanized and rural sites per location in our analysis. Shaded in grey, are the sites where parotoid gland secretions were sampled.

Site	GPS coordinates (Latitude, Longitude)	Location	Urban	n_{Female}	n_{Male}
Amatikulu	-29.110000, 31.600000	Durban	no	3	1
Botanical Garden	-29.847000, 31.005972	Durban	yes	21	23
Durban North	-29.778111, 31.020917	Durban	yes	4	2
Lychee Farm	-29.469500, 31.228333	Durban	no	9	11
Shongweni	-29.860361, 30.721139	Durban	no	18	9
UKZN Campus	-29.817667, 30.943583	Durban	yes	34	25
Brise Fer	-20.383000, 57.422833	Mauritius	no	5	4
Le Pouce Mt.	-20.190722, 57.517417	Mauritius	no	8	2
Mt. Camizard	-20.329861, 57.700611	Mauritius	no	19	14
Notre Dame	-20.143194, 57.556917	Mauritius	yes	16	7
Palma Cave	-20.111333, 57.718528	Mauritius	yes	3	2
Savanne	-20.479000, 57.481028	Mauritius	no	19	14
Vacoas	-20.290000, 57.500000	Mauritius	yes	29	36
Étang du Gol	-21.285806, 55.388028	Réunion	yes	3	2
Grand Etang	-21.094944, 55.655778	Réunion	no	33	32
La Saline les Bains	-21.085743, 55.233047	Réunion	yes	25	6
Sainte-Rose	-21.129111, 55.792667	Réunion	yes	10	36
Constantia	-34.024278, 18.422972	Cape Town	yes	45	45

Table S2. Model summary of a linear mixed effects model investigating the effect of urbanization of the habitat on the relative size of the parotoid gland. The table gives coefficient estimates (*β*) that are representative of log_{10}-transformed morphological measures with their corresponding standard errors (*SE*) and *t*-values. Variance estimates (σ^2) are supplied for residuals and random effects. All significant values ($p < 0.05$) are bolded. Reference levels for each categorical variable are supplied in parentheses following the variable name.

**Variable Names**				
Fixed Effects	*β*	*SE*	*t*	*p*
**Intercept (Cape Town, Rural, Female)**	**-0.901**	**0.132**	**-6.823**	**<0.001**
**log_{10} SVL**	**1.708**	**0.068**	**24.940**	**<0.001**
Location (Durban)	0.054	0.049	1.101	0.297
**Location (Mauritius)**	**-0.225**	**0.050**	**-4.535**	**0.001**
**Location (Réunion)**	**-0.137**	**0.050**	**-2.753**	**0.021**
**Sex (Male)**	**-0.057**	**0.009**	**-6.532**	**<0.001**
Urbanization (Urban)	0.002	0.025	0.064	0.950
Random Effects	σ^2			
Intercept (Site)	0.002			
Residuals	0.010			

Table S3. Average proportions of identified bufadienolides in the parotoid gland secretions. This table shows the tentatively identified bufadienolide compounds in the parotoid gland secretions sampled with their average proportions in % ($\bar{x}$ %) and their corresponding standard errors (*SE*%). The compounds are represented by a unique identifier (Toxin ID) and a tentative compound name is also provided. Information on the adduct, retention time in min (t_R), mass-to-charge-ratio (*m/z*), formula, mass error in ppm and MS^E fragments is also provided. Lastly, references for tentative identification (if available) are given.

Toxin ID	$\bar{x}$%	*SE*%	Compound	Adduct	t_R (min)	*m/z*	Formula	Mass Error (ppm)	MS^E Fragments	Reference
Toxin 1	0.6	0.063	O-pimelate-l-argininyl-Arenobufagin/Bufarenogin/Hellebrigenin	$[M+H]^+$	4.54	731.3839	$C_{37}H_{55}N_4O_{11}$	-3.8	333.1769	
Toxin 2	0.3	0.070	O-pimelate-l-argininyl-Arenobufagin/Bufarenogin/Hellebrigenin or pimeroyl-l-argininyl-Hydroxyhellebrigenin	$[M+H]^+$	4.65	731.3853	$C_{37}H_{55}N_4O_{11}$	-1.9	co-elution[a]	[1]
Toxin 3	2.1	0.253	O-suberate-l-argininyl-Arenobufagin/Bufarenogin/Hellebrigenin	$[M+H]^+$	4.71	745.4005	$C_{38}H_{57}N_4O_{11}$	-2.5	347.1912	
Toxin 4	1.0	0.164	O-suberate-l-argininyl-Arenobufagin/Bufarenogin/Hellebrigenin	$[M+H]^+$	4.86	745.4007	$C_{38}H_{57}N_4O_{11}$	-2.3	347.19	
Toxin 5	1.1	0.136	O-suberate-l-argininyl-Marinobufagin/Resibufaginol/deacetylCinobufagin	$[M+H]^+$	5.16	729.4045	$C_{38}H_{57}N_4O_{10}$	-4.1	347.1931	
Toxin 6	0.8	0.166	O-suberate-l-argininyl-Marinobufagin/Resibufaginol/deacetylCinobufagin	$[M+H]^+$	5.26	729.405	$C_{38}H_{57}N_4O_{10}$	-3.4	347.1924	
Toxin 7	3.2	0.203	O-pimelate-l-argininyl-Bufalin	$[M+H]^+$	5.43	701.4124	$C_{37}H_{57}N_4O_9$	-0.3	333.1769	
Toxin 8	1.1	0.102	diO-suberate-l-argininyl-Bufogenin/Bufalone /3,14-Dihydroxybufa-4,20,22-trienolide	$[M+H]^+$	5.56	729.4033	$C_{38}H_{57}N_4O_{10}$	-5.8	363.1867	
Toxin 9	11.1	0.706	O-suberate-l-argininyl-Bufalin	$[M+H]^+$	5.62	715.4689	$C_{38}H_{59}N_4O_9$	1	347.1914	
Toxin 10	13.5	0.509	O-pimelate-l-argininyl-Bufogenin /Bufalone /3,14-	$[M+H]^+$	5.79	699.3964	$C_{37}H_{55}N_4O_9$	-0.7	333.1763	

			Dihydroxybufa-4,20,22-trienolide							
Toxin 11	45.1	0.932	O-suberate-l-argininyl-Bufogenin/Bufalone/3,14-Dihydroxybufa-4,20,22-trienolide	$[M+H]^+$	5.97	713.4129	$C_{38}H_{57}N_4O_9$	0.4	347.1926	
Toxin 12	0.5	0.101	Resibufaginol / Marinobufagin / deacetylCinobufagin	$[M+H]^+$	6.2	401.2324	$C_{24}H_{33}O_5$	-1	tr[b]	1-3
Toxin 13	1.2	0.140	O-azelate-l-argininyl-Bufogenin/Bufalone/3,14-Dihydroxybufa-4,20,22-trienolide	$[M+H]^+$	6.28	727.4285	$C_{39}H_{59}N_4O_9$	0.4	361.2086	1
Toxin 14	4.1	0.316	Bufalin	$[M+H]^+$	6.59	387.2529	$C_{24}H_{35}O_4$	-1.5	369.2413,351.2322	1-3
Toxin 15	14.3	0.995	Bufogenin/Bufalone/3,14-Dihydroxybufa-4,20,22-trienolide	$[M+H]^+$	7.15	385.2378	$C_{24}H_{33}O_4$	-0.3	367.225	1-3

[a]Fragment ions not clear due to co-elution with other compound(s)

[b]Fragment ions detected only at trace levels

Table S4. Outcome of a linear model investigating differences in compound evenness (Shannon diversity) between locations and sexes. (A) The table gives coefficient estimates (*β*) with their corresponding standard errors (*SE*) and *t*-values. All significant values (p < 0.05) are bolded. Reference levels for each categorical variable are supplied in parentheses following the variable name. (B) Post-hoc multiple comparisons of bufadionolide evenness between locations. All *p*-values were Tukey-adjusted to facilitate multiple comparisons.

(A) Model summary				
Variable Names	*β*	*SE*	*t*	*p*
**Intercept (Durban, Female)**	**1.790**	**0.035**	**51.294**	**<0.001**
Location (Cape Town)	-0.054	0.040	-1.356	0.184
Location (Mauritius)	-0.037	0.042	-0.890	0.380
**Location (Réunion)**	**0.087**	**0.040**	**2.147**	**0.039**
**Sex (Male)**	**-0.148**	**0.028**	**-5.264**	**<0.001**

(B) Multiple comparisons between locations				
Contrasts	*β*	*SE*	*t*	P_{adj}
Durban vs. Cape Town	0.054	0.040	1.356	0.535
Durban vs. Mauritius	0.037	0.042	0.890	0.810
Durban vs. Réunion	-0.087	0.040	-2.147	0.158
Cape Town vs. Mauritius	-0.017	0.038	-0.446	0.970
**Cape Town vs. Réunion**	**-0.141**	**0.037**	**-3.822**	**0.003**
**Mauritius vs. Réunion**	**-0.124**	**0.037**	**-3.309**	**0.011**

Table S5. Post-hoc multiple comparisons investigating differences in compound proportions. Presented are odds ratios with corresponding standard errors (*SE*), *z*-values, and Tukey-adjusted *p*-values for each comparison. Significant contrasts were bolded. The results are averaged for location and sex.

Contrasts	*odds ratio*	*SE*	*z*	p_{adj}
**Toxin 1 vs. Toxin 2**	**2.280**	**0.372**	**5.041**	**<0.001**
**Toxin 1 vs. Toxin 3**	**0.334**	**0.039**	**-9.488**	**<0.001**
Toxin 1 vs. Toxin 4	0.855	0.117	-1.141	0.998
**Toxin 1 vs. Toxin 5**	**0.560**	**0.070**	**-4.617**	**<0.001**
Toxin 1 vs. Toxin 6	0.986	0.145	-0.094	1
**Toxin 1 vs. Toxin 7**	**0.182**	**0.023**	**-13.405**	**<0.001**
**Toxin 1 vs. Toxin 8**	**0.547**	**0.066**	**-4.997**	**<0.001**
**Toxin 1 vs. Toxin 9**	**0.050**	**0.006**	**-23.628**	**<0.001**
**Toxin 1 vs. Toxin 10**	**0.038**	**0.004**	**-32.296**	**<0.001**
**Toxin 1 vs. Toxin 11**	**0.007**	**0.001**	**-49.599**	**<0.001**
Toxin 1 vs. Toxin 12	1.440	0.226	2.307	0.585
**Toxin 1 vs. Toxin 13**	**0.521**	**0.065**	**-5.236**	**<0.001**
**Toxin 1 vs. Toxin 14**	**0.145**	**0.018**	**-15.414**	**<0.001**
**Toxin 1 vs. Toxin 15**	**0.039**	**0.005**	**-26.959**	**<0.001**
**Toxin 2 vs. Toxin 3**	**0.147**	**0.021**	**-13.377**	**<0.001**
**Toxin 2 vs. Toxin 4**	**0.375**	**0.060**	**-6.141**	**<0.001**
**Toxin 2 vs. Toxin 5**	**0.246**	**0.038**	**-9.047**	**<0.001**
**Toxin 2 vs. Toxin 6**	**0.433**	**0.077**	**-4.685**	**<0.001**
**Toxin 2 vs. Toxin 7**	**0.080**	**0.011**	**-17.887**	**<0.001**
**Toxin 2 vs. Toxin 8**	**0.240**	**0.037**	**-9.367**	**<0.001**
**Toxin 2 vs. Toxin 9**	**0.022**	**0.003**	**-27.318**	**<0.001**
**Toxin 2 vs. Toxin 10**	**0.017**	**0.002**	**-30.566**	**<0.001**
**Toxin 2 vs. Toxin 11**	**0.003**	**0.000**	**-43.996**	**<0.001**
Toxin 2 vs. Toxin 12	0.631	0.119	-2.445	0.481
**Toxin 2 vs. Toxin 13**	**0.228**	**0.034**	**-9.933**	**<0.001**
**Toxin 2 vs. Toxin 14**	**0.064**	**0.010**	**-17.214**	**<0.001**
**Toxin 2 vs. Toxin 15**	**0.017**	**0.003**	**-25.754**	**<0.001**
**Toxin 3 vs. Toxin 4**	**2.560**	**0.290**	**8.294**	**<0.001**
**Toxin 3 vs. Toxin 5**	**1.680**	**0.170**	**5.074**	**<0.001**
**Toxin 3 vs. Toxin 6**	**2.950**	**0.391**	**8.168**	**<0.001**
**Toxin 3 vs. Toxin 7**	**0.545**	**0.051**	**-6.473**	**<0.001**
**Toxin 3 vs. Toxin 8**	**1.640**	**0.161**	**5.023**	**<0.001**
**Toxin 3 vs. Toxin 9**	**0.148**	**0.014**	**-20.443**	**<0.001**
**Toxin 3 vs. Toxin 10**	**0.114**	**0.008**	**-32.555**	**<0.001**
**Toxin 3 vs. Toxin 11**	**0.021**	**0.001**	**-60.582**	**<0.001**
**Toxin 3 vs. Toxin 12**	**4.300**	**0.635**	**9.883**	**<0.001**
**Toxin 3 vs. Toxin 13**	**1.560**	**0.152**	**4.544**	**<0.001**
**Toxin 3 vs. Toxin 14**	**0.435**	**0.048**	**-7.627**	**<0.001**
**Toxin 3 vs. Toxin 15**	**0.115**	**0.012**	**-20.51**	**<0.001**
**Toxin 4 vs. Toxin 5**	**0.655**	**0.081**	**-3.409**	**0.047**
Toxin 4 vs. Toxin 6	1.150	0.174	0.943	1
**Toxin 4 vs. Toxin 7**	**0.213**	**0.025**	**-13.066**	**<0.001**
**Toxin 4 vs. Toxin 8**	**0.640**	**0.079**	**-3.637**	**0.022**

**Toxin 4 vs. Toxin 9**	**0.058**	**0.007**	**-24.032**	**<0.001**
**Toxin 4 vs. Toxin 10**	**0.044**	**0.004**	**-31.525**	**<0.001**
**Toxin 4 vs. Toxin 11**	**0.008**	**0.001**	**-49.491**	**<0.001**
Toxin 4 vs. Toxin 12	1.680	0.280	3.114	0.113
**Toxin 4 vs. Toxin 13**	**0.609**	**0.074**	**-4.093**	**0.004**
**Toxin 4 vs. Toxin 14**	**0.170**	**0.023**	**-13.14**	**<0.001**
**Toxin 4 vs. Toxin 15**	**0.045**	**0.006**	**-23.474**	**<0.001**
**Toxin 5 vs. Toxin 6**	**1.760**	**0.234**	**4.269**	**0.002**
**Toxin 5 vs. Toxin 7**	**0.325**	**0.039**	**-9.345**	**<0.001**
Toxin 5 vs. Toxin 8	0.977	0.108	-0.209	1
**Toxin 5 vs. Toxin 9**	**0.088**	**0.011**	**-19.668**	**<0.001**
**Toxin 5 vs. Toxin 10**	**0.068**	**0.006**	**-33.052**	**<0.001**
**Toxin 5 vs. Toxin 11**	**0.013**	**0.001**	**-52.753**	**<0.001**
**Toxin 5 vs. Toxin 12**	**2.570**	**0.413**	**5.858**	**<0.001**
Toxin 5 vs. Toxin 13	0.930	0.105	-0.646	1
**Toxin 5 vs. Toxin 14**	**0.260**	**0.034**	**-10.181**	**<0.001**
**Toxin 5 vs. Toxin 15**	**0.069**	**0.009**	**-20.876**	**<0.001**
**Toxin 6 vs. Toxin 7**	**0.185**	**0.029**	**-10.805**	**<0.001**
**Toxin 6 vs. Toxin 8**	**0.555**	**0.075**	**-4.381**	**0.001**
**Toxin 6 vs. Toxin 9**	**0.050**	**0.008**	**-18.735**	**<0.001**
**Toxin 6 vs. Toxin 10**	**0.039**	**0.005**	**-27.82**	**<0.001**
**Toxin 6 vs. Toxin 11**	**0.007**	**0.001**	**-41.374**	**<0.001**
Toxin 6 vs. Toxin 12	1.460	0.249	2.203	0.663
**Toxin 6 vs. Toxin 13**	**0.528**	**0.075**	**-4.48**	**<0.001**
**Toxin 6 vs. Toxin 14**	**0.147**	**0.022**	**-12.81**	**<0.001**
**Toxin 6 vs. Toxin 15**	**0.039**	**0.006**	**-22.478**	**<0.001**
**Toxin 7 vs. Toxin 8**	**3.000**	**0.333**	**9.906**	**<0.001**
**Toxin 7 vs. Toxin 9**	**0.271**	**0.015**	**-23.793**	**<0.001**
**Toxin 7 vs. Toxin 10**	**0.209**	**0.017**	**-19.304**	**<0.001**
**Toxin 7 vs. Toxin 11**	**0.039**	**0.003**	**-45.228**	**<0.001**
**Toxin 7 vs. Toxin 12**	**7.890**	**1.240**	**13.12**	**<0.001**
**Toxin 7 vs. Toxin 13**	**2.860**	**0.283**	**10.587**	**<0.001**
Toxin 7 vs. Toxin 14	0.798	0.089	-2.032	0.78
**Toxin 7 vs. Toxin 15**	**0.211**	**0.023**	**-14.02**	**<0.001**
**Toxin 8 vs. Toxin 9**	**0.090**	**0.010**	**-21.643**	**<0.001**
**Toxin 8 vs. Toxin 10**	**0.070**	**0.006**	**-33.055**	**<0.001**
**Toxin 8 vs. Toxin 11**	**0.013**	**0.001**	**-55.201**	**<0.001**
**Toxin 8 vs. Toxin 12**	**2.630**	**0.382**	**6.647**	**<0.001**
Toxin 8 vs. Toxin 13	0.951	0.104	-0.457	1
**Toxin 8 vs. Toxin 14**	**0.266**	**0.029**	**-12.209**	**<0.001**
**Toxin 8 vs. Toxin 15**	**0.070**	**0.007**	**-25.636**	**<0.001**
Toxin 9 vs. Toxin 10	0.769	0.064	-3.185	0.093
**Toxin 9 vs. Toxin 11**	**0.142**	**0.010**	**-27.26**	**<0.001**
**Toxin 9 vs. Toxin 12**	**29.100**	**4.500**	**21.776**	**<0.001**
**Toxin 9 vs. Toxin 13**	**10.500**	**1.030**	**23.979**	**<0.001**
**Toxin 9 vs. Toxin 14**	**2.940**	**0.306**	**10.357**	**<0.001**
Toxin 9 vs. Toxin 15	0.779	0.081	-2.391	0.521
**Toxin 10 vs. Toxin 11**	**0.185**	**0.006**	**-55.694**	**<0.001**
**Toxin 10 vs. Toxin 12**	**37.800**	**5.270**	**26.047**	**<0.001**

**Toxin 10 vs. Toxin 13**	**13.700**	**1.110**	**32.291**	**<0.001**
**Toxin 10 vs. Toxin 14**	**3.820**	**0.380**	**13.491**	**<0.001**
Toxin 10 vs. Toxin 15	1.010	0.096	0.141	1
**Toxin 11 vs. Toxin 12**	**204.000**	**27.900**	**38.962**	**<0.001**
**Toxin 11 vs. Toxin 13**	**74.000**	**5.740**	**55.434**	**<0.001**
**Toxin 11 vs. Toxin 14**	**20.700**	**1.910**	**32.719**	**<0.001**
**Toxin 11 vs. Toxin 15**	**5.470**	**0.482**	**19.3**	**<0.001**
**Toxin 12 vs. Toxin 13**	**0.362**	**0.056**	**-6.536**	**<0.001**
**Toxin 12 vs. Toxin 14**	**0.101**	**0.012**	**-18.875**	**<0.001**
**Toxin 12 vs. Toxin 15**	**0.027**	**0.003**	**-31.296**	**<0.001**
**Toxin 13 vs. Toxin 14**	**0.279**	**0.033**	**-10.783**	**<0.001**
**Toxin 13 vs. Toxin 15**	**0.074**	**0.009**	**-22.56**	**<0.001**
**Toxin 14 vs. Toxin 15**	**0.265**	**0.013**	**-26.916**	**<0.001**

Table S6. Post-hoc multiple comparisons investigating differences in compound proportions between locations. Presented are odds ratios with corresponding standard errors (*SE*), *z*-values, and Tukey-adjusted *p*-values for each comparison. Significant contrasts are bolded. The results are averaged over sex.

**Contrasts**				
Toxin 1	*odds ratio*	*SE*	*z*	p_{adj}
Durban vs. Cape Town	1.344	0.408	0.975	1
Durban vs. Mauritius	0.994	0.304	-0.021	1
Durban vs. Réunion	0.6	0.167	-1.836	1
Cape Town vs. Mauritius	0.739	0.212	-1.056	1
Cape Town vs. Réunion	0.447	0.116	-3.115	0.807
Mauritius vs. Réunion	0.604	0.147	-2.072	1
Toxin 2				
Durban vs. Cape Town	0.885	0.376	-0.287	1
Durban vs. Mauritius	0.613	0.265	-1.132	1
**Durban vs. Réunion**	**0.151**	**0.056**	**-5.099**	**0.001**
Cape Town vs. Mauritius	0.693	0.261	-0.973	1
**Cape Town vs. Réunion**	**0.171**	**0.0542**	**-5.567**	**<0.001**
**Mauritius vs. Réunion**	**0.246**	**0.0746**	**-4.625**	**<0.001**
Toxin 3				
Durban vs. Cape Town	1.401	0.299	1.58	1
Durban vs. Mauritius	0.768	0.158	-1.281	1
Durban vs. Réunion	0.483	0.0906	-3.881	0.178
Cape Town vs. Mauritius	0.548	0.105	-3.141	0.788
**Cape Town vs. Réunion**	**0.345**	**0.0607**	**-6.047**	**<0.001**
Mauritius vs. Réunion	0.629	0.0954	-3.059	0.847
Toxin 4				
Durban vs. Cape Town	0.883	0.282	-0.388	1
Durban vs. Mauritius	0.873	0.289	-0.409	1
**Durban vs. Réunion**	**0.185**	**0.0514**	**-6.074**	**<0.001**
Cape Town vs. Mauritius	0.989	0.286	-0.04	1
**Cape Town vs. Réunion**	**0.209**	**0.0483**	**-6.777**	**<0.001**
**Mauritius vs. Réunion**	**0.212**	**0.0488**	**-6.73**	**<0.001**
Toxin 5				
Durban vs. Cape Town	2.479	0.666	3.377	0.578
Durban vs. Mauritius	1.201	0.311	0.705	1
Durban vs. Réunion	1.243	0.31	0.874	1
Cape Town vs. Mauritius	0.484	0.131	-2.678	0.985
Cape Town vs. Réunion	0.502	0.131	-2.637	0.990
Mauritius vs. Réunion	1.035	0.245	0.147	1
Toxin 6				
Durban vs. Cape Town	0.905	0.32	-0.283	1
Durban vs. Mauritius	1.254	0.473	0.6	1
Durban vs. Réunion	0.345	0.115	-3.188	0.750
Cape Town vs. Mauritius	1.386	0.466	0.971	1
Cape Town vs. Réunion	0.381	0.111	-3.321	0.631
**Mauritius vs. Réunion**	**0.275**	**0.0825**	**-4.301**	**0.042**
Toxin 7				

Durban vs. Cape Town	0.976	0.184	-0.128	1
Durban vs. Mauritius	1.198	0.239	0.906	1
Durban vs. Réunion	1.328	0.26	1.445	1
Cape Town vs. Mauritius	1.227	0.226	1.114	1
Cape Town vs. Réunion	1.36	0.245	1.706	1
Mauritius vs. Réunion	1.108	0.205	0.553	1
Toxin 8				
Durban vs. Cape Town	1.025	0.218	0.119	1
Durban vs. Mauritius	1.609	0.388	1.973	1
Durban vs. Réunion	1.116	0.248	0.495	1
Cape Town vs. Mauritius	1.569	0.349	2.023	1
Cape Town vs. Réunion	1.089	0.219	0.421	1
Mauritius vs. Réunion	0.694	0.156	-1.628	1
Toxin 9				
Durban vs. Cape Town	1	0.18	0.002	1
Durban vs. Mauritius	1.16	0.219	0.785	1
Durban vs. Réunion	1.75	0.325	3.01	0.877
Cape Town vs. Mauritius	1.16	0.201	0.856	1
Cape Town vs. Réunion	1.75	0.298	3.288	0.662
Mauritius vs. Réunion	1.509	0.261	2.375	1
Toxin 10				
Durban vs. Cape Town	1.034	0.12	0.289	1
Durban vs. Mauritius	1.038	0.126	0.303	1
Durban vs. Réunion	0.984	0.116	-0.133	1
Cape Town vs. Mauritius	1.003	0.112	0.03	1
Cape Town vs. Réunion	0.952	0.103	-0.456	1
Mauritius vs. Réunion	0.949	0.104	-0.48	1
Toxin 11				
Durban vs. Cape Town	0.938	0.0778	-0.767	1
Durban vs. Mauritius	1.058	0.0918	0.648	1
Durban vs. Réunion	1.07	0.0902	0.807	1
Cape Town vs. Mauritius	1.127	0.0894	1.512	1
Cape Town vs. Réunion	1.141	0.0876	1.715	1
Mauritius vs. Réunion	1.012	0.079	0.151	1
Toxin 12				
Durban vs. Cape Town	1.618	0.58	1.343	1
Durban vs. Mauritius	1.519	0.539	1.176	1
Durban vs. Réunion	1.337	0.463	0.837	1
Cape Town vs. Mauritius	0.938	0.323	-0.184	1
Cape Town vs. Réunion	0.826	0.29	-0.544	1
Mauritius vs. Réunion	0.88	0.291	-0.386	1
Toxin 13				
Durban vs. Cape Town	0.846	0.158	-0.897	1
**Durban vs. Mauritius**	**2.994**	**0.747**	**4.396**	**0.029**
Durban vs. Réunion	1.695	0.369	2.427	0.999
**Cape Town vs. Mauritius**	**3.538**	**0.823**	**5.431**	**<0.001**
Cape Town vs. Réunion	2.004	0.395	3.528	0.437
Mauritius vs. Réunion	0.566	0.138	-2.339	1
Toxin 14				

Durban vs. Cape Town	1.033	0.216	0.157	1
Durban vs. Mauritius	0.773	0.164	-1.212	1
Durban vs. Réunion	1.062	0.223	0.286	1
Cape Town vs. Mauritius	0.748	0.145	-1.496	1
Cape Town vs. Réunion	1.028	0.197	0.143	1
Mauritius vs. Réunion	1.374	0.262	1.668	1
Toxin 15				
Durban vs. Cape Town	0.788	0.153	-1.229	1
Durban vs. Mauritius	0.577	0.116	-2.74	0.975
Durban vs. Réunion	0.668	0.131	-2.057	1
Cape Town vs. Mauritius	0.732	0.133	-1.717	1
Cape Town vs. Réunion	0.848	0.15	-0.931	1
Mauritius vs. Réunion	1.159	0.207	0.825	1

Table S7. Post-hoc multiple comparisons investigating differences in compound proportions between sexes. Presented are odds ratios with corresponding standard errors (*SE*), *z*-values, and Tukey-adjusted *p*-values for each comparison. Significant contrasts were bolded. The results are averaged over location.

Compound ID	*Contrasts*	*odds ratio*	*SE*	*z*	p_{adj}
Toxin 1	Female vs. Male	1.395	0.285	1.633	0.102
Toxin 2	Female vs. Male	1.218	0.293	0.818	0.414
**Toxin 3**	**Female vs. Male**	**1.406**	**0.191**	**2.511**	**0.012**
Toxin 4	Female vs. Male	1.35	0.243	1.667	0.096
Toxin 5	Female vs. Male	0.902	0.168	-0.554	0.580
Toxin 6	Female vs. Male	1.209	0.275	0.836	0.403
Toxin 7	Female vs. Male	1.209	0.166	1.387	0.166
Toxin 8	Female vs. Male	0.908	0.143	-0.61	0.542
Toxin 9	Female vs. Male	1.167	0.15	1.2	0.230
**Toxin 10**	**Female vs. Male**	**0.823**	**0.0677**	**-2.367**	**0.018**
**Toxin 11**	**Female vs. Male**	**0.793**	**0.0465**	**-3.957**	**<0.001**
**Toxin 12**	**Female vs. Male**	**1.702**	**0.433**	**2.091**	**0.037**
Toxin 13	Female vs. Male	1.228	0.187	1.349	0.178
Toxin 14	Female vs. Male	1.265	0.183	1.619	0.106
Toxin 15	Female vs. Male	1.288	0.174	1.868	0.0617

Table S8. Potential vertebrate predators of Guttural Toads in each study location. Predators have been grouped in taxonomic groups (non-avian reptiles, birds, and mammals). For each species, the common name is followed by the scientific name and the reference to a possible predation on a toad is provided. Predator species within a taxonomic group are presented in no specific order. Asterisks (*) are provided behind species that are considered invasive in the given location. Please note that this table does not provide an exhaustive overview of all predators of Guttural Toads in each study location. Rather, this table indicates known toad predator species in each location and thereby, to the best of our knowledge gives an overview of the predator community (i.e., types of vertebrate predator) in a location in relation to the other locations.

Location	Predator species
**Durban**	*Non-avian reptiles*<br>Puff Adder (*Bitis arietans*) [4]<br>Cape Cobra (*Naja nivea*) [5]<br>Mozambique Spitting Cobra (*Naja mossambica*) [5]<br>Snouted Cobra (*Naja annulifera*) [5]<br>Cape File Snake (*Limaformosa capensis*) [6]<br>Rhombic Night Adder (*Causus rhombeatus*) [7]<br>Herald Snake (*Crotaphopeltis hotamboeia*) [8]<br>Brown Forest Cobra (*Naja subfulva*) [8]<br>Rinkhals (*Hemachatus haemachatus*) [8]<br>Rock Monitor (*Varanus albigularis*) [9]<br>Nile Monitor (*Varanus niloticus*) [9]<br><br>*Birds*<br>Hamerkop (*Scopus umbretta*) [8]<br>Black-Chested Snake Eagle (*Circaetus pectoralis*) [10]<br>Southern Fiscal Shrike (*Lanius collaris*) [11]<br>Grey Heron (*Ardea cinerea*) [11]<br>Barn Owl (*Tyto alba*) [12]<br>House Crow (*Corvus splendens*) [13]<br>Pied Crow (*Corvus albus*) [13]<br>Verreaux's Eagle-Owl (*Ketupa lactea*) [14]<br><br>*Mammals*<br>African Clawless Otter (*Aonyx capensis*) [15]<br>Spotted-Necked Otter (*Hydrictis maculicollis*) [15]<br>Brown Rat (*Rattus norvegicus*) [16]*<br>Feral Pig (*Sus scrofa*) [17]*<br>Feral Cat (*Felis catus*) [18]*<br>Banded Mongoose (*Mungos mungo*) [19]<br>Water Mongoose (*Atilax paludinosus*) [19]
**Cape Town**	*Non-avian reptiles*<br>Puff Adder (*Bitis arietans*) [4]<br>Cape Cobra (*Naja nivea*) [5]<br>Herald Snake (*Crotaphopeltis hotamboeia*) [8]<br>Rinkhals (*Hemachatus haemachatus*) [8]<br><br>*Birds*<br>House Crow (*Corvus splendens*) [13]<br>Pied Crow (*Corvus albus*) [13]<br>Cape crow (*Corvus capensis*) [13]<br>Southern Fiscal Shrike (*Lanius collaris*) [11]<br>Grey Heron (*Ardea cinerea*) [11]

	Barn Owl (*Tyto alba*) [12]<br>Verreaux's Eagle-Owl (*Ketupa lactea*) [14]<br><br>*Mammals*<br>African Clawless Otter (*Aonyx capensis*) [15]<br>Brown Rat (*Rattus norvegicus*) [16*]<br>Feral Cat (*Felis catus*) [18*]
**Mauritius**	*Non-avian reptiles*<br>Indian Wolf Snake (*Lycodon aulicus*) [20*]<br><br>*Birds*<br>House Crow (*Corvus splendens*) [13*]<br><br>*Mammals*<br>Brown Rat (*Rattus norvegicus*) [16*]<br>Black Rat (*Rattus rattus*) [16*]<br>Asian House Shrew (*Suncus murinus*) [21*]<br>Feral Cat (*Felis catus*) [18*]<br>Small Indian Mongoose (*Urva auropunctata*) [22*]<br>Feral Pig (*Sus scrofa*) [17*]
**Réunion**	*Non-avian reptiles*<br>Indian Wolf Snake (*Lycodon aulicus*) [20*]<br><br>*Birds*<br>House Crow (*Corvus splendens*) [13*]<br><br>*Mammals*<br>Brown Rat (*Rattus norvegicus*) [16*]<br>Black Rat (*Rattus rattus*) [16*]<br>Asian House Shrew (*Suncus murinus*) [21*]<br>Feral Cat (*Felis catus*) [18*]

References

1. Schmeda-Hirschmann G, de Andrade JP, Soto-Vasquez MR, Alvarado-García PAA, Palominos C, Fuentes-Retamal S, et al. The parotoid gland secretion from Peruvian toad *Rhinella horribilis* (Wiegmann, 1833): Chemical composition and effect on the proliferation and migration of lung cancer cells. Toxins. 2020;12: 608.

2. Meng Q, Yau L-F, Lu J-G, Wu Z-Z, Zhang B-X, Wang J-R, et al. Chemical profiling and cytotoxicity assay of bufadienolides in toad venom and toad skin. Journal of ethnopharmacology. 2016;187: 74–82.

3. Cao Y, Wu J, Pan H, Wang L. Chemical profile and multicomponent quantitative analysis for the quality evaluation of toad venom from different origins. Molecules. 2019;24: 3595.

4. Glaudas X, Kearney TC, Alexander GJ. Museum specimens bias measures of snake diet: a case study using the ambush-foraging puff adder (*Bitis arietans*). Herpetologica. 2017;73: 121–128.

5. Shine R, Branch WR, Webb JK, Harlow PS, Shine T, Keogh JS. Ecology of cobras from southern Africa. Journal of Zoology. 2007;272: 183–193.

6. Shine R, Branch WR, Harlow PS, Webb JK. Sexual dimorphism, reproductive biology, and food habits of two species of African filesnakes (*Mehelya*, Colubridae). Journal of Zoology. 1996;240: 327–340.

7. Deans R. *Causus rhombeatus* (Lichtenstein, 1823) Diet. African Herp News. 2011;54: 14–15.

8. Maritz RA, Maritz B. Sharing for science: high-resolution trophic interactions revealed rapidly by social media. PeerJ. 2020;8: e9485.

9. Dalhuijsen K, Alexander GJ, Branch WR. A comparative analysis of the diets of *Varanus albigularis* and *Varanus niloticus* in South Africa. African Zoology. 2014;49: 83–93.

10. del Hoyo J, Elliott A, Sargatal J, Cabot J. Handbook of the Birds of the World: Jacamars to Woodpeckers. Lynx Edicions; 1992.

11. Hockey PA, Dean WRJ, Ryan PG, Maree S, Brickman BM. Roberts' birds of southern Africa. Trustees of the John Voelcker Bird Book Fund; 2005.

12. Datta SC. Enriched school environment for the effective bio-activity of barn owls. International journal of Horticulture, Agriculture and Food science(IJHAF) 2019d. 2019;3: 119–126.

13. Vogrin M, Vogrin N. Hooded crow *Corvus cornix* takes a common toad Bufo bufo bufo. Ornis Svecica. 1998;8: 42–44.

14. Avery G, Robertson AS, Palmer NG, Prins AJ. Prey of Giant Eagle Owls in the De Hoop Nature Reserve, Cape Province, and some observations on hunting strategy. Ostrich. 1985;56: 117–122. doi:10.1080/00306525.1985.9639579

25. Butler JRA, Du Toit JT. Diet and conservation status of Cape clawless otters in eastern Zimbabwe. South African Journal of Wildlife Research. 1994;24: 41–47.

16. Marshall BM, Casewell NR, Vences M, Glaw F, Andreone F, Rakotoarison A, et al. Widespread vulnerability of Malagasy predators to the toxins of an introduced toad. Current Biology. 2018;28: R654–R655.

17. Ballari SA, Barrios-García MN. A review of wild boar *Sus scrofa* diet and factors affecting food selection in native and introduced ranges. Mammal Review. 2014;44: 124–134.

18. Woods M, McDonald RA, Harris S. Predation of wildlife by domestic cats *Felis catus* in Great Britain. Mammal review. 2003;33: 174–188.

19. Smithers RH. Smithers Mammals of Southern Africa. Penguin Random House South Africa; 2012.

20. Hossain MS, Saeed MA, Ahsan MF, Jaman MF, Chayan HAR, Hasan S, et al. Present status, challenges and prospects of snake farming in Bangladesh. Bangladesh Journal of Zoology. 2022;50: 121–133.

21. Gautam B, Bhattarai S. Predation on the Indian Bull frog *Hoplobatrachus tigerinus* (Daudin, 1802)(Anura: Dicroglossidae) by the Asian House Shrew *Suncus murinus* (Linnaeus, 1766)(Soricomorpha: Soricidae) from Nepal. Journal of Animal Diversity. 2020;2: 37–41.

22. Gaubert P, Angelici FM, Ćirović D. Small Indian Mongoose *Urva auropunctata* (Hodgson, 1836). Handbook of the Mammals of Europe. Springer; 2024. pp. 1–19.
